# Electrical stimulation of the nucleus basalis of meynert: a systematic review of preclinical and clinical data

**DOI:** 10.1038/s41598-021-91391-0

**Published:** 2021-06-03

**Authors:** Muhammad Nazmuddin, Ingrid H. C. H. M. Philippens, Teus van Laar

**Affiliations:** 1 Department of Neurology, Parkinson Expertise Center, University Medical Center Groningen, University of Groningen, Hanzeplein 1 AB-51, 9700RB Groningen, The Netherlands; 2 Animal Science Department, Biomedical Primate Research Centre (BPRC), P.O. Box 3306, 2280 GH Rijswijk, The Netherlands

**Keywords:** Dementia, Cognitive ageing

## Abstract

Deep brain stimulation (DBS) of the nucleus basalis of Meynert (NBM) has been clinically investigated in Alzheimer’s disease (AD) and Lewy body dementia (LBD). However, the clinical effects are highly variable, which questions the suggested basic principles underlying these clinical trials. Therefore, preclinical and clinical data on the design of NBM stimulation experiments and its effects on behavioral and neurophysiological aspects are systematically reviewed here. Animal studies have shown that electrical stimulation of the NBM enhanced cognition, increased the release of acetylcholine, enhanced cerebral blood flow, released several neuroprotective factors, and facilitates plasticity of cortical and subcortical receptive fields. However, the translation of these outcomes to current clinical practice is hampered by the fact that mainly animals with an intact NBM were used, whereas most animals were stimulated unilaterally, with different stimulation paradigms for only restricted timeframes. Future animal research has to refine the NBM stimulation methods, using partially lesioned NBM nuclei, to better resemble the clinical situation in AD, and LBD. More preclinical data on the effect of stimulation of lesioned NBM should be present, before DBS of the NBM in human is explored further.

## Introduction

The nucleus basalis of Meynert (NBM), a cholinergic nucleus in the basal forebrain, provides extensive projections to all cortical areas and is one of the targets of deep brain stimulation (DBS) in Alzheimer’s disease (AD) and both Lewy Body dementia (LBD) subtypes, including Parkinson’s disease dementia (PDD) and dementia with Lewy bodies (DLB)^1–3^. The importance of cortical cholinergic input from the NBM on cognitive processing, including attentional processes, and its defects in AD and LBD have been extensively reviewed^4–6^. Therefore, electrical stimulation of cholinergic NBM neurons has been hypothesized to upregulate its efferent projections and to alleviate the associated symptomatology^7,8^.

However, so far the clinical results of NBM DBS are quite poor and inconclusive. NBM DBS in one patient with PDD seemed to improve ideomotor apraxia and global cognitive functions, but these findings were obscured by additional bilateral stimulation of the subthalamic nucleus for his motor symptoms^9,10^. Another study showed that NBM DBS alleviated visual hallucinations in two PDD patients while effects on cognitive functioning assessed by neuropsychological testing varied in AD and DLB patients^1–3^.

This article provides a systematic review of the preclinical and clinical evidence of the behavioral and neurophysiological effects of the NBM DBS, in order to identify potential pitfalls and gaps in implementing the therapy in clinical practice.

## Results

**Figure 1:**
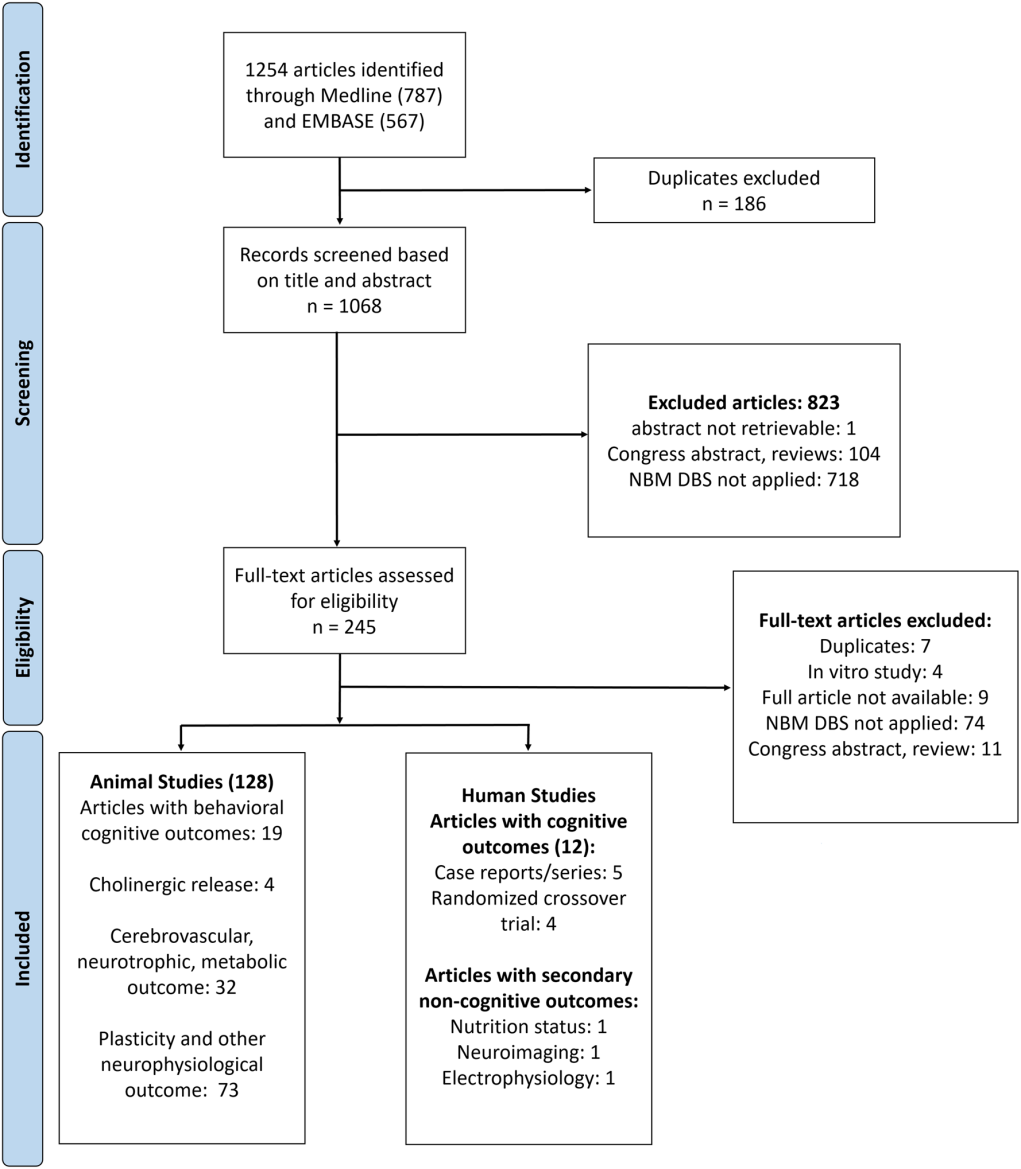
Diagram illustrating the search strategy of the systematic review.

We retrieved 787 articles from PubMed and 567 articles from Embase based on the predefined search strategy. Overall, 1068 articles remained, after having removed duplicates and finally 128 preclinical studies and 12 clinical reports were included according to our eligibility criteria. The included animal studies were further grouped according to their primary outcomes (Fig. 1) and will be described accordingly in this section. In summary, 19 studies reported behavioral outcomes after NBM stimulation, 4 studies reported the stimulation effect on cortical acetylcholine (ACh) release, 32 articles reported the changes of the cerebral blood flow (CBF), cerebral metabolism, and neurotrophic release upon NBM DBS. Finally, 73 articles reported the effect of NBM DBS on neuroplasticity and cortical-subcortical connectivity based on electrophysiology measures. Finally, a comparative overview of the current clinical findings on NBM DBS is discussed.

### Preclinical Evidence of NBM DBS

#### Effects of NBM DBS on behavioral and cognitive performance

We classified the studies investigating the effect of NBM DBS on cognitive performance into three subcategories based on the availability of a relevant control group and comparisons made in the studies as presented in Table 1.

**Table 1:** Characteristics of animal studies with information on the effect of NBM DBS on behavioral cognitive outcomes.

No	Author, year	Species, strain, sex	Age	Comparisons	Bilateral/unilateral stimulation (right/left)	Stimulation parameter	Behavioral task	Stimulation timing	Stimulation duration
**I. Sham vs NBM stimulation (rodent study)**									
1	Montero-Pastor et al. 2001^11^	Rats, Wistar, male	92.8 (SEM = 0.66) days old	Sham vs stimulation	Unilateral (right)	Intermittent stimulation (500 ms with electrical pulses and 500 ms without electrical pulses), 100 Hz, 500 µs, 60–90 µA	Two-way active avoidance paradigm	Immediately after acquisition training	20 min
2	Montero-Pastor et al. 2004^12^	Rats, Wistar, male	97.63 (SD = 5.52) days old	Sham vs stimulation	Unilateral (right)	Intermittent stimulation (500 ms with electrical pulses and 500 ms without electrical pulses), 100 Hz, 500 µs, 100 µA	Two-way active avoidance paradigm	Immediately before (a) or after (b) acquisition training, or before 24-h-retention assessment (c)	30–45 min
3	Boix-Trelis et al. 2006^15^	Rats, Wistar, male	98.23 (SEM = 0.78) days old	Sham vs stimulation	Unilateral (left/right)*	Intermittent stimulation (500 ms with electrical pulses and 500 ms without electrical pulses), 100 Hz, 500 µs, 100 µA	Relational odor-association task—the social transmission of food preference (STFP)	Before acquisition training	20 min
4	Reed et al. 2011^16^	Rats	N/A	Sham vs stimulation	Unilateral (right)	Intermittent stimulation (electrical pulses was synchronized to the cue presentation), 20 pulses at 100 Hz, 100 µs, 120–200 µA, biphasic	Tone discrimination task	During acquisition training	3 h/day for 20 days
5	Lee et al. 2016^13^	Rats, Sprague–Dawley, male	6 weeks old	NBM-intact vs NBM lesioned vs NBM-lesioned + electrode implant vs NBM-lesioned + stimulation	Unilateral (right)	120 Hz, 90 μs, 1 V	Morris water maze	Before acquisition training	1 h/day; 7 days in total
6	Huang et al. 2018^14^	C57/BL6-Tg APP/P1 transgenic mice, Wild type WT C57BL/6 mice, male	4, 6, 9, 12 months old	Control non-surgical mice, sham stimulation, stimulation	Unilateral (left)	Continuous stimulation, (10/50/100/130) Hz, 90 μs, 1 A	Morris water maze	Before acquisition training	60 min per day for 30 days; 60 min per day for 7, 14, 21, 28 days; 60 min per day for 21 days
**II. Unsynchronized vs synchronized stimulation (rodent study)**									
7	McLin III et al. 2002^17^	Rats, Wistar, male	N/A	Synchronized vs unsynchronized stimulation	Unilateral (right)	100 Hz bipolar, pulse width is unknown, 50–100 μA, 200 ms train duration	Classical conditioning**	During acquisition training	N/A
8	Weinberger et al. 2006^20^	Rats, Sprague–Dawley, male	115 (SD = 33) days old	Weak amplitude + unsychronized vs weak amplitude + synchronized stimulation; moderate amplitude + unsynchronized vs moderate + synchronized	Unilateral (right)	bipolar, 100 Hz, 200 µs, 65.7 ± 9.0 µA (moderate)/46.7 ± 12.1µA (weak), 200 ms train duration, biphasic pulses	Classical conditioning**		1–3 training sessions, ~ 4 h per session
9	Miasnikov et al. 2006^19^	Rats, Sprague–dawley, male	110 ± 24 days	Synchronized vs unsynchronized stimulation	Unilateral (right)	bipolar, 100 Hz, 200 µs, ~ 66 µA, 200 ms train duration, biphasic pulses	Classical conditioning**		1 training session, ~ 4 h per session
10	Miasnikov et al. 2009^22^	Rats, Sprague–Dawley, male	112 (SD = 24) days old	Synchronized vs unsynchronized stimulation	Unilateral (right)	bipolar, 100 Hz, 200 µs, ~ 66 µA, 200 ms train duration, biphasic pulses	Classical conditioning**		1 training sessions, ~ 4 h per session
11	Weinberger et al. 2009^18^	Rats, Sprague–Dawley, male	N/A	Synchronized vs unsynchronized stimulation	Unilateral (right)	bipolar, 100 Hz, 200 µs, ~ 66 µA, 200 ms train duration, biphasic pulses	Classical conditioning**		3 training sessions, ~ 4 h per session
12	Miasnikov et al. 2011^21^	Rats, Sprague–Dawley	92 (SD = 7) days	Synchronized vs unsynchronized stimulation	Unilateral (right)	bipolar, 100 Hz, 200 µs, ~ 66 µA, 200 ms train duration, biphasic pulses	Classical conditioning**		3 training sessions, ~ 4 h per session
**III. Single-group, repeated-measures study with non-human primates & rodents**									
13	Miasnikov et al. 2008a^25^	Rats, Sprague–Dawley, male	104 (SD = 17) days old	Before vs after stimulation	Unilateral (right)	100 Hz, 200 µs, 66 µA, 200 ms train duration, biphasic pulses	Classical conditioning**	During cognitive assessment	1 training sessions, ~ 4 h per session
14	Miasnikov et al. 2008^26^	Rats, Sprague–Dawley, male	N/A	Before vs after stimulation	Unilateral (right)	100 Hz, 200 µs, 66 µA, 200 ms train duration, biphasic pulses	Classical conditioning**		1 training sessions, ~ 4 h per session
15	Avila & Lin 2014^27^	Rats, Long-Evans, male	3–6 months	Stimulation vs no stimulation	Bilateral	100 µs, 11 pulses with 10 ms interstimulus interval, 16–48 µA, biphasic pulses	Auditory-cued discrimination task		N/A
16	Mayse et al. 2015^28^	Rats, Long-Evans, male	6 months	Stimulation vs no stimulation	Bilateral	1–3 pulses at 100 Hz, 24–48 µA, 100 µs, biphasic pulses	Stop no-reward task		N/A
17	Liu et al. 2017^23^	Rhesus monkey (*M. mulata*)	6 years old	Continuous vs intermittent vs no stimulation	Bilateral	Continuous stimulation, 80 Hz, 100 μs, 200 uA, biphasic pulses; Intermittent stimulation (20-s ON, 40-s OFF), 60 Hz, 100 μs, 200 μA, biphasic pulses	Delayed match-to-sample task		N/A
18	Liu et al. 2018^24^	Rhesus monkey (*M. mulata*), male	6 years old	Intermittent vs no stimulation	Bilateral	Intermittent stimulation (20-s ON, 40-s OFF), 60 Hz, 100 us, 200 μA, biphasic pulses	Continuous performance task		N/A
19	Koulousakis et al. 2020^29^	Tg APP/P1 transgenic rats, male	18 months	Continuous vs intermittent vs no stimulation	Bilateral	Intermittent stimulation (20-s ON, 40-s OFF), positive monophasic pulses, 100 µs, 200 µA, 60 Hz; continuous stimulation, 20 Hz	Modified Barnes maze task		N/A

N/A data is not available. *left or right NBM, the number within the group was made. **sound of 8-kHz tone as the conditioned stimulus, NBM stimulation as the unconditioned stimulus, and respiratory change index as the conditioned response balance.

The first group consists of six studies involving comparisons between animals with NBM DBS and sham stimulation^11–16^. These articles reported in total eight independent experiments using mainly male rats, with the age varying between 6–14 weeks. NBM DBS was performed unilaterally, predominantly at the right-sided NBM. Electrical pulses were delivered either in intermittent or continuous fashion in experiments of single or multiple sessions of 20–60 min, and varying frequencies (10–130 Hz) for 1 to 30 days. Cognitive tasks included the passive avoidance test, the social transmission of food preference, the tone-discrimination task, and the Morris water maze test. Endpoints focused on memory function, including encoding, consolidation, and retrieval. The significant positive effect of NBM DBS was reported on the encoding and immediate retention of memory, but not on the long-term retention of memory^11,12^. The trend of a positive effect was similarly found on the learning speed of the tone discrimination task and the spatial memory test based on the Morris water maze test.

The second subgroup consists of six studies which focused on the timing of electrical pulses to the NBM, using a classical conditioning paradigm^17–22^. All the experiments were performed by a single research group, using male rats of 12–17 weeks old. The DBS electrode was implanted unilaterally in the right NBM. The electrode position was verified afterwards histologically. An in-house-developed learning and memory task was used. The disruption of ongoing respiratory pattern, immediately after repetitive exposure to the sound stimuli, was considered as a behavioral marker of memory. Electrical stimulation of the NBM was able to associate the frequency of tone as the conditioned stimulus to the change of respiratory behavior. This associative behavior started 24 h after training and persisted for two weeks after the stimulation.

The last subgroup consists of five rodent studies and two studies with non-human primates with a single-group repeated-measure design^23–29^. The studies with rhesus macaques consisted of a longitudinal observation on the efficacy of bilateral electrical NBM stimulation with continuous and intermittent fashion delivered in different frequencies. The cognitive effects, specifically on working memory and attention, were compared. Individual-based analysis showed the significant improvement of the intermittent stimulation approach which delivered biphasic electrical pulses of 60/80 Hz to the NBM for 20 s, interleaved with 40-s non-stimulation. On the other hand, continuously stimulation, both at 20 and 80 Hz, worsened the behavioral endpoints. A complementary study performed in rodents confirmed the potential benefit of intermittent stimulation for cognition, specifically related to spatial memory, as assessed by the modified Barnes maze.

**Figure 2:**
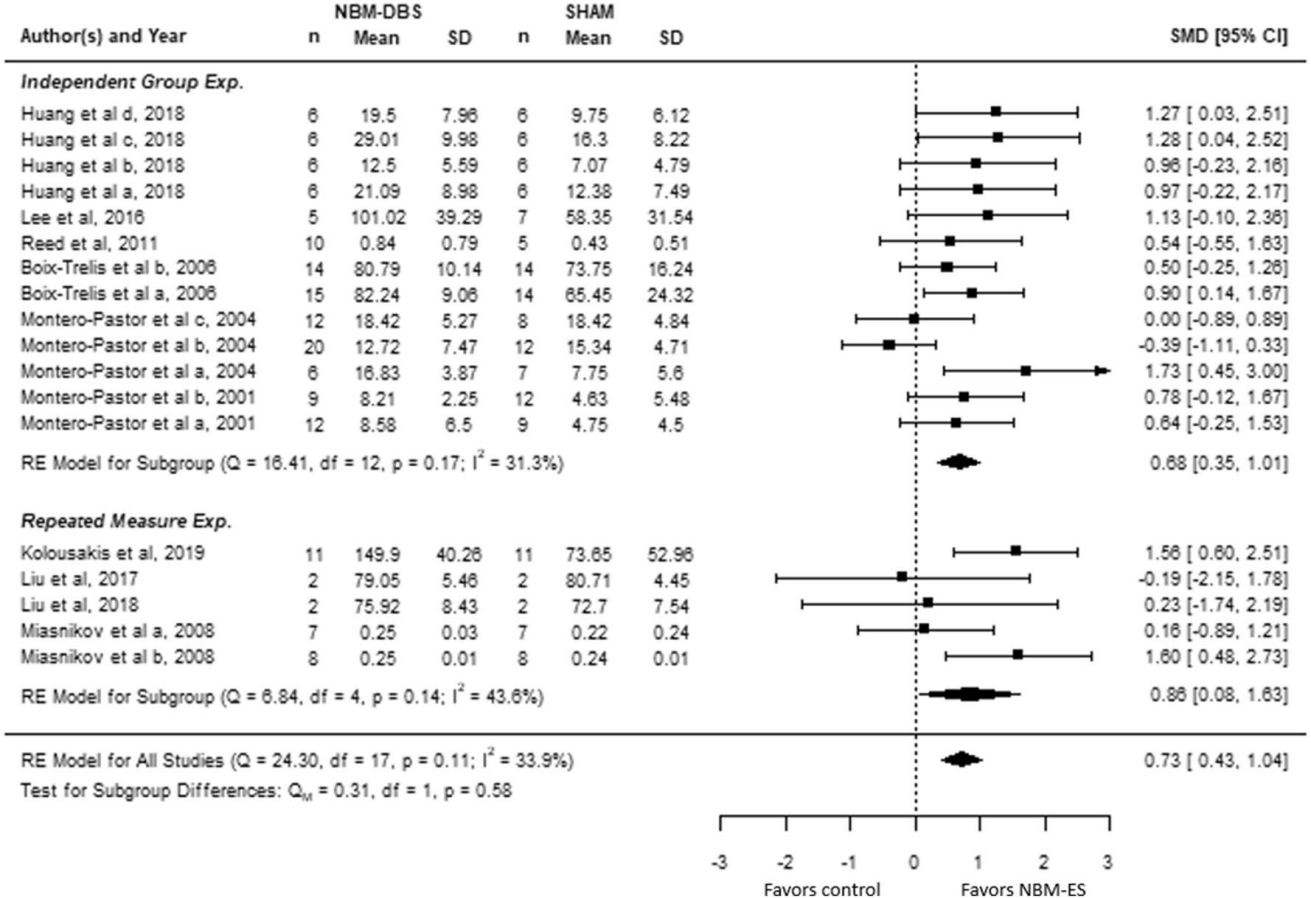
The forest plot summarizes the effect size and its 95% CIs of NBM DBS on behavioral cognitive performance in studies with repeated measure design and independent-control design separately, and combined.

Results of the quantitative analysis of studies with repetitive-measure and sham-control designs are showed by a forest plot (Fig. 2). In sham-controlled studies, NBM DBS significantly improved cognitive performance by 0.68 SMD (95% CI 0.35–1.01). The heterogeneity between sham-controlled studies was not significant (Q = 16.41; *df* = 12*; I*^2^ = 31.3%*; P* = 0.17)*.* Similarly, studies with repetitive-measure design showed the positive effect of NBM DBS on animal’s cognitive performance (SMD = 0.86, 95% CI 0.08–1.63) and non-significant between-study heterogeneity (Q = 6.84; *df* = 4*; I*^2^ = 43.6%*; P* = 0.14). The pooled effect size of all size was 0.73 (95% CI 0.43–1.04) and the heterogeneity among pooled studies remained non-significant (Q = 24.3; *df* = 4*; I*^2^ = 33.9%*; P* = 0.11). Funnel plot analysis indicated asymmetry of the estimated effect size, suggesting potential publication bias. Two studies were subsequently imputed by trim-and-fill analysis (Supplementary Fig. 1). The positive effect of NBM DBS on cognitive performance remained significant after the correction (SMD = 0.64, 95% CI 0.33–0.94).

The methodological quality of studies with behavioral outcomes was summarized in Supplementary Table 1, 2 and 3. Randomization during animal grouping was mentioned but not in sufficient details in all studies. Explanations regarding randomization and blinding during experimental procedures were also missing. However, the blinding during the outcome assessment were reported in half of the studies involving sham-stimulation group. Two studies with sham-controlled design did not report the verification of electrode placement in the NBM^14,16^. The reporting of stimulation parameters was found incomplete in four sham-controlled studies.

#### Effects of NBM DBS on cortical ACh release

Four studies in rodents investigated the effect of electrical stimulation of the NBM on the cortical release of ACh (Supplementary Table 4). All studies, except one, used anesthetized animals. All studies were uncontrolled longitudinal observations. The applied stimulation parameters varied in patterns, frequencies, pulse width, and amplitudes. The release of ACh was measured by collecting the interstitial substance of the frontoparietal cortex every 10 to 20 min prior to, during, and after stimulation of the NBM. Acetylcholine concentrations were analyzed using gas chromatographic-mass spectrometry or with high-performance liquid chromatography.

All studies showed that NBM DBS, both continuously and intermittently, enhanced the release of ACh in the cortex^30–33^. Low-frequency stimulation (20–50 Hz) showed a greater effect on cortical ACh release compared to high-frequency stimulation (100–200 Hz)^33^. However, another study reported a better effect of high-frequency compared to low-frequency stimulation when electrical pulses were delivered through a shorter burst of 10 s in a relatively higher stimulation amplitude (2000 µA) compared to other studies^31^.

#### Effects on cerebral blood flow and metabolism

Thirty-two studies in rodents explored the effect of NBM DBS on cerebrovascular functions (Supplementary Table 5). Autoradiography, helium clearance/mass spectrometry, laser Doppler and speckle flowmetry were performed to measure changes of cerebral blood flow (CBF) and vascular structures. NBM DBS enhanced cortical and subcortical blood flow, applied either as single-burst, continuous- or phasic stimulation, with stimulation periods up to 1 h. Especially the parenchymal vessels were dilated by activating the muscarinic and nicotinic receptors at the inner arteriole layer. Pial arteries, receiving cholinergic input from the internal carotid ganglia, remained unaffected.

Stimulation parameters, ageing, electrode positioning, and additional treatment such as sustained nicotinic subcutaneous infusion proved to modulate the vasodilatory response induced by NBM DBS^34–37^. The effects were amplitude-dependent^32,35–38^. Electrical pulses up to 100 Hz and train duration up to 10–30 s had a maximum effect on CBF and did not show further increase of the CBF beyond these values. All studies used a relatively long pulse width at 500 µs.

#### Effects on cortical and subcortical plasticity and connectivity

The final categories of studies in relation with NBM DBS comprises electrophysiological studies examining the NBM anatomical connectivity with other brain structures as well as scrutinizing the effect of NBM activation on subcortical and cortical activities and plasticity (Supplementary Table 6). The immediate, short-, and long-term consequences of stimulation were investigated by applying single-train and repetitive stimulation. Studies investigating the facilitatory impact of NBM DBS on cortical plasticity were typically designed by concomitantly presenting sensory cues or electrical stimulation of corresponding subcortical and cortical areas, while delivering electrical pulses to the NBM^39–42^. Overall, activation of NBM neurons by electrical stimulation poses deterministic excitatory and synaptic modulatory effects to the innervated brain areas, resulting in behavioral consequences, including the facilitation of learning and memory processes.

NBM DBS was able to excite its projected neurons and triggered EEG activation indicated by enhanced gamma oscillatory activity^43–46^. Its post-synaptic modulatory effects were shown in several studies. Burst NBM DBS, concurrently applied either with electrical stimulation to the medial as well as the lateral geniculate nucleus of the thalamus (MGN or LGN) and with tactile stimuli to facial whisker, enhanced the thalamocortical synaptic output of the corresponding projected cortices^47–49^. NBM DBS facilitated frequent action potentials triggered by EPSPs and inhibited persistent spontaneous hyperpolarization, which outlasted several seconds post-stimulation in the auditory cortex^50^. Furthermore, the amplification effect was observed to be dependent from muscarinic stimulation, and was also more pronounced with presynaptic input such as stimulation of the contralateral LGN or tactile stimuli to non-principle whisker^49^. The observed neuronal potentiation at single-unit recording coincided with the enhancement of evoked field potentials during electrocorticography, indicating synchronous activation of neural populations in the NBM-innervated cortices^47,49^. Finally, the behavioral impact of NBM DBS-elicited potentiation was demonstrated by the potentiation of vibrissae muscle contractions, induced by electrical pulses to the motor cortex, following single-burst NBM DBS^41^.

The facilitatory role of NBM DBS in brain plasticity was shown by the combination of NBM DBS with variable sensory cues like tone frequency, tone amplitude and visual orientation, lead to increasing neural firing, expansion of tonotopic representation in the cortical receptive field and its downstream structures, and to the promotion of task learning as a behavioral consequence^16,39,48,51,51–57^. The increased neural firing and tonotopic expansion are observed within 1 h after 40 pairing trials and gradually renormalized 24 h post-stimulation^51,52^. Another study including a high trial repetition (~ 300/session) and a longer conditioning period (20 days), showed persistent receptive plasticity 20 days after the last conditioning, which disappeared at day 40. Notably, the behavioral performance remained stable despite the renormalized tonotopic map observed at this time point^16^.

### Clinical studies of NBM DBS

Nine primary clinical studies, including five case reports and four randomized crossover studies, involving patients with mild-to-moderate AD, PDD and DLB were identified. The description concerning subject characteristics, stimulation parameters, and the main finding of these studies are summarized in Table 2. In addition, two other studies explored the effect of NBM DBS on non-behavioral aspects, including nutritional status and brain evoked potentials. Finally, one study reported the potential structural neuroimaging correlates of NBM DBS efficacy.

**Table 2:** Characteristics and results of NBM DBS clinical studies reporting cognitive outcomes.

No	Author, year	Study design	Patient diagnosis, number, age	Bilateral / unilateral	Stimulation parameter	Mentioned additional treatment	Study duration with active NBM DBS	Results
1	Turnbull et al., 1985^58^	Case report	AD, 1, 74 years	Unilateral (Left)	Intermittent (15-s-ON & 12-min-OFF); 50 Hz, 3 V, 210 ms	Not reported	9 months	the decline of the cortical glucose metabolism after 9 months was smaller in the ipsilateral than the contralateral hemisphere
2	Freund et al., 2009^10^	Case report	PDD, 1, 71 years	Bilateral	Continuous, 20 Hz, 120 µs, 1.0 V	DBS of the subthalamic nucleus, dopaminergic medication with levodopa equivalent dose 312.5 mg/day	13 weeks	scores in neuropsychological testing improved during stimulation and worsened after one week without NBM stimulation
3	Barnikol et al., 2010^9^	Case report	PDD, 1, 71 years	Bilateral	Continuous, 20 Hz, 120 µs, 1.0 V	DBS of the subthalamic nucleus	16 months	NBM DBS significantly improved apraxia symptoms
4	Kuhn et al., 2015a^1^	Double-blind crossover study (2 weeks ON-2 weeks OFF), followed by 48-week open label study	AD, 6, 57–79 years	Bilateral	Continuous, 10/20 Hz, 90–150 µs, 2.0–4.5 V	Galantamine, Mirtazapine, Donepezil, Lorazepam, Memantine, Escitalopram (combination varried across patients)	50 weeks	changes (improvement and worsening) of cognitive performance based on neuropsychological testing varried across patients, cerebral glucose metabolism increased in three patients
5	Kuhn et al., 2015b^94^	Case report	AD, 2, 61 & 67 years	Bilateral	Continuous, 20 Hz, pulse width and amplitude were not reported	Not reported	26–28 months	general neuropsychological testing using ADAS-Cog showed stable or improved results while fluctuative results were observed in the other tests
6	Gratwicke et al., 2018^2^	Double-blind crossover study	PDD, 6, 65.2 [10.7] years	Bilateral	Continuous, 20 Hz, 60 µs, 1.5–3 V	Dopaminergic medication, Rivastigmine, Citalopram, Fesoteradine, Quetiapine, Venlafaxine	6 weeks	cognitive performance was insignificantly different between ON and OFF DBS state. Statistically significant improvement of hallucination subscale of the NPI was observed in NBM DBS state which was driven by the results of two patients
7	Nombela et al., 2019^62^	Case report	PD-MCI, 1, 68 years	Bilateral	Continuous, 20 Hz, 60 µs, 2 mA	DBS of the internal pallidum	3 months	Improvement in spatial memory test (ROCF), two out of four tests in executive function (TMT-A and subscale similarities of WAIS IV), and a phonological verbal fluency for letter P, compared to baseline
8	Gratwicke et al., 2020^3^	Double-blind crossover study	DLB, 6, 65–75 years	Bilateral	Continuous, 20 Hz, 60 µs, 2–3.5 V	Dopaminergic medication, Rivastigmine, Donepezil, Citalopram, Sertraline, Amytriptylline, Clonazepam, Venlafaxine	6 weeks	Group-wise, no significant difference was observed between NBM and sham stimulation. The neuropsychiatric complains reduced in 3/5 patients
9	Maltête et al., 2020^61^	Double-blind crossover study	DLB, 6, 50–69 years	Bilateral	Continuous, 20/50/100 Hz, 60–90 µs, 2.5–3 V	Rivastigmine	3 months	No significant difference on cognitive performance between sham versus NBM DBS

ADAS-Cog Alzheimer’s Disease Assessment Scale Cognitive Subscale, ROCF Rey-Osterrieth Complex Figure, TMT-A Trail Making Test A, WAIS IV Wechsler Adult Intelligence Scale IV.

The very first clinical NBM DBS experiment was reported in 1985, presenting a 71-year-old AD patient, who received unilateral NBM stimulation for nine months^58^. Intermittent electrical stimulation (15-s ON/12-min OFF) at 50 Hz was applied. Cortical glucose metabolism was less reduced in the stimulated hemisphere, compared to baseline. However, cognitive functioning remained unchanged.

After more than two decades, another clinical study reported about the simultaneous bilateral placement of DBS electrodes in the NBM and STN, the standard clinical DBS target in PDD^10^. The patient was a 71-year-old man with PDD, and received high-frequency stimulation of the STN for three months, before the low-frequency NBM DBS was switched ON. Turning ON the STN DBS improved motor function significantly, but hardly changed cognitive function. However, turning ON NBM DBS improved attention, alertness and drive, whereas these functions significantly worsened after switching OFF the NBM stimulator. Additional report of the same patient similarly showed remarkable improvement of his apraxic symptoms as systematically measured by the Florida apraxia screening test. This improvement was reflected when performing daily activities^9^.

Four randomized, controlled crossover studies comprising each six mild-to-moderate AD, PDD, or DLB did not show significant differences in cognitive performance between sham vs. NBM DBS. An open-label stimulation parameter screening based on subjective and/or objective clinical assessment was applied, either before the subjects entered the crossover study in studies with PDD and DLB or after in AD studies. The continuous low-frequency (10–50 Hz) stimulation was chosen except for one DLB subject who received the continuous high-frequency (100 Hz) stimulation. Monopolar stimulation using either of the two most distal contacts was applied. The pulse width was set between 60 and 150 µs with the amplitude ranging at 1.5 to 4.5 V.

A longitudinal follow-up after one year in the study involving AD patients showed that the worsening of cognitive performance occurred in two patients who had poor baseline scores, while the other patients showed stable or improved performance^1^. The group’ nutrition status after one year was unchanged^59^. Furthermore, secondary outcome measures with FDG PET indicated a significant positive correlation between the changes in cognitive performance and the glucose utilization around the active electrodes. Additionally, the preserved cortical thickness in several fronto-parieto-temporal regions as examined with patients’ preoperative MRI was associated with stable cognitive capacity at one-year follow up. Finally, the electrophysiological effects of NBM DBS were studied by means of evoked potentials, using the passive auditory oddball paradigm. This study showed normalization of P50-N100 after NBM DBS^60^.

Three other crossover studies on NBM DBS were applied in DLB or PDD. Decreased neuropsychiatric inventory (NPI) scores, which was mainly driven by a decrease of visual hallucination, apathy and caregiver distress, were observed in two PDD and three DLB subjects^2,3^. Additionally, significant improvement of functional connectivity in the frontoparietal and default mode network in DLB subjects were noticed^3^. In another study, alleviation of motor symptoms and increased metabolic activity at the superior lingual gyrus following NBM DBS was shown while cognitive function remained unchanged^61^.

Finally, a recent case report showed the use of a novel DBS system in one PD patient with mild cognitive impairment (PD-MCI), providing simultaneous high-frequency stimulation of the internal part of the globus pallidus (GPi) and low-frequency stimulation of the NBM, via a single eight-contact lead. However, the cognitive effect of bilateral low frequency stimulation of the NBM, in addition to stimulation of the GPi, was inconclusive^62^.

## Discussion

**Figure 3:**
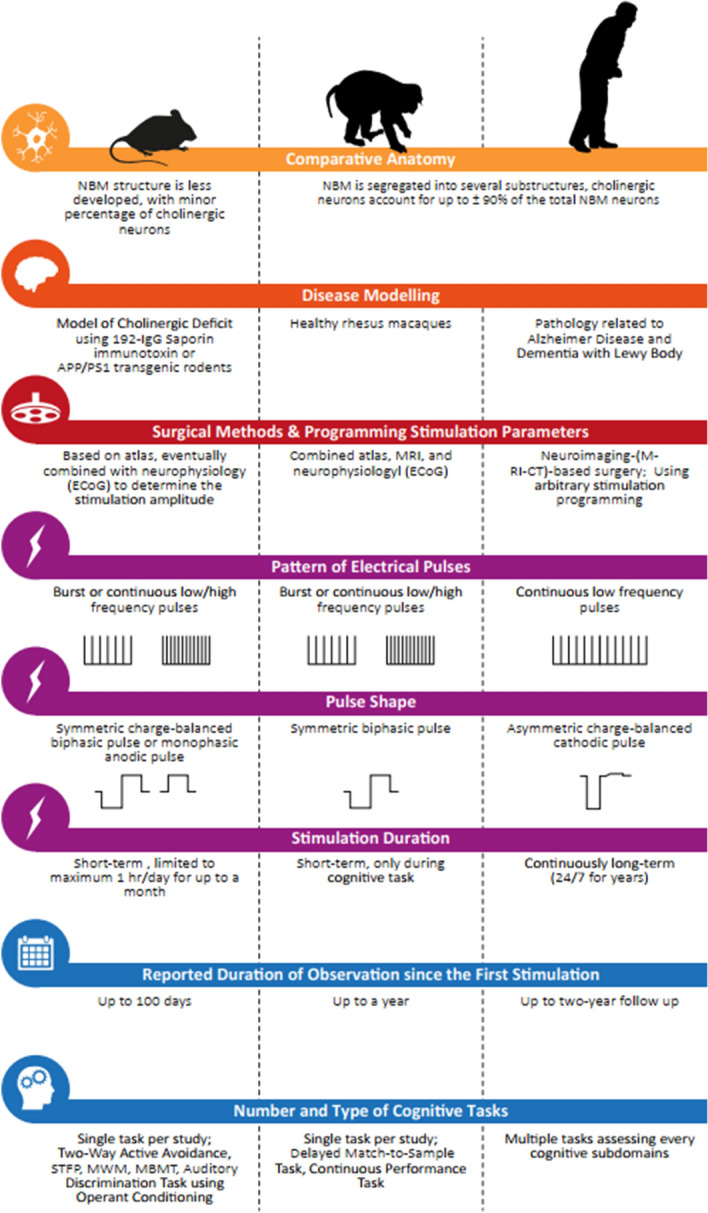
Differences in the anatomy of cholinergic basal forebrain, the presence of AD/LBD pathology, surgical and stimulation methodology, duration of stimulation and observation, and cognitive measure approach between rodent, non-human primate, and clinical studies investigating the cognitive efficacy of NBM DBS.

This study systematically reviewed and compared the current preclinical and clinical evidence on the effects of electrical NBM stimulation, including behavioral and neurophysiological effects. Here we will discuss the major findings as illustrated in Fig. 3 and the consequences for future clinical research on NBM DBS.

Our meta-analysis identified the overall positive effect of NBM DBS on attention, working memory, learning and long-term memory, using both negative and positive reinforcement. The positive behavioral impact is supported by the potential of NBM stimulation to improve cortical and subcortical plasticity, to induce cortical vasodilation and to enhance the release of ACh and neuroprotective factors, without necessarily increasing the metabolic activity of the projecting area. This means that NBM DBS has a great potential, which is very much dependent from the stimulation paradigms and surgical approaches.

This review showed that intrinsic animal characteristics such as age, baseline cognitive capacity and the intactness of the NBM directly correlate to the cognitive benefit of NBM stimulation. Similarly, NBM DBS studies in AD patients indicated a better effect in younger patients, likely reflecting the more intact cholinergic functioning in this subgroup. This finding is in accordance with other studies showing that the cholinergic treatment response is predicted by the degree of functional cholinergic integrity, based on resting state functional MRI and cholinergic PET scans in patients with MCI^63,64^. However, another recent study in early AD patients did not find a significant correlation between the basal forebrain volume and the response of acetylcholinesterase inhibitor after six months of treatment^65^. So, more data is needed to solve this issue, because this should be the basis of selection for future clinical studies with NBM DBS. Possibly only patients at the MCI stage are suitable for NBM DBS, but this is unclear at this moment.

In animal studies, incorporation of intraoperative electrocorticography (ECoG) to observe cortical desynchronization elicited by NBM DBS leads to precise electrode implantation and improves efficacy in cognition and neuroplasticity^18,24,25^. On the other hand, the current surgical approach in clinical studies still depends on the stereotactic surgical map and high-resolution structural imaging in their targeting protocol. Meanwhile, the use of ECoG within clinical DBS studies is emerging, not only merely for neurophysiological research in the intraoperative setting, but also as a neural-machine interface for close-loop DBS system in epilepsy and movement disorders^66–71^. In the context of NBM DBS, ECoG would be helpful to assist in the stereotactic targeting, and also to guide the optimization of stimulation parameters based on the cortical desynchronization. ECoG, in combination with behavioral monitoring, can be used to monitor the chronic effects of NBM DBS and to guide the refinement of stimulation parameters. Additionally, it will also lead to ECoG-based biomarkers which can be integrated in closed-loop technology. Quantitative EEG to assess oscillatory changes in cortical areas might be an alternative for ECoG, to refine stimulation parameters and the steering of electrodes^67,72,73^.

The efficacy of NBM DBS is primarily dependent from optimal stimulation parameters. Current clinical studies used continuous low-frequency stimulation (10–50 Hz) to stimulate the NBM. The low frequency is based on the observed firing rates of the NBM in rodents during movements, and on the simplified hypothesis that low-frequency pulses exerts excitatory effects, while high-frequency pulses would have inhibitory effects^10,74^. Current understanding of the DBS-related mechanisms of action however points beyond this simplified excitatory-inhibitory hypothesis^75,76^. Another issue is related to the continuity of stimulation; intermittent vs. continuous stimulation. Recent animal studies in healthy rhesus macaques and APP/PS1 transgenic rats that systematically compared the cognitive effects of continuous versus intermittent burst of 20-s, 60-Hz, ON and 40-s OFF stimulation indicated the superior benefit of the intermittent stimulation on working memory^23,24^. Additionally, the majority of rodent studies of NBM DBS applied the micro-burst (in 500 ms-ON and 500-ms-OFF) stimulation.

The translational interpretation of NBM-related animal data should be made with caution, due to several issues. The first issue is related to the anatomical difference of the cholinergic nuclei between rodents and primates, including the balance between cholinergic and non-cholinergic neurons in the basal forebrain. Also, the difference in the structural segregation and the cortical projections from NBM subdivisions between rodents and human has significant consequences for the cognitive and behavioral effects^77–79^. For example, in human, the anterior NBM projects its neurons more to the frontal lobe whereas the posterior part innervates the temporal region^80,81^. Meanwhile in rodent, frontocaudal organization determines the distinct cortical layer projections^82^. Furthermore, several studies pointed specifically that the volume loss of the posterior NBM is associated with decreased global cognitive capacity and memory retrieval in AD and PD patients^83,84^. Thus, where the electrode is placed within the NBM becomes crucial in driving the clinical outcomes. Considering the rostrocaudal elongation and projection of the NBM, one may propose to stereotactically target the NBM periorbitally to provide as many DBS contacts as possible to the whole NBM.

An important issue is that animal studies are based on relatively short stimulation periods in contrast with the continuous 24-h stimulation in clinical studies. Typically, the electrical pulses were applied prior to, during, or after behavioral training. None of the animal studies systematically compared the effect of different durations of stimulation and the timing of stimulation within 24 h. The timing seems to be very important, as shown by the fact that if NBM DBS is applied during sleep, the normal sleep wake cycle is disrupted, which is important for the physiological process of learning and memory^85,86^.

Another translational obstacle is that most preclinical data were derived from unilateral stimulations of non-diseased rodents, with intact basal forebrains. Only three studies with rodents addressed this issue. The first one used intraventricular 192-IgG Saporin infusion to lesion the NBM, which was stimulated thereafter^13^, while the others used transgenic mouse and rat models of AD, overexpressing Amyloid-β precursor and Presenilin1 protein (APP/PS1)^14,29^. However, only the PS1 study conducted bilateral NBM stimulation, whereas no systematic comparison between unilateral versus bilateral stimulation was reported. This is an important flaw, because of the lateralization of the basal forebrain and the NBM function in attentional processing^87–89^.

Furthermore, several animal studies applied stimulation amplitudes that either hardly induced neuronal excitability (± 1 µA) or likely induced tissue damage (1 mA), rather than stimulating neuronal tissues. Additionally, monophasic stimulation was mostly used, instead of the biphasic pulses that are normally used in clinical NBM DBS.

Finally, several animal studies reported incomplete stimulation parameters, without mentioning pulse length, frequency or pulse shape, which have great impact on the final outcomes^90–95^. Despite the methodological problems to translate the current preclinical evidence into clinical practice, at least three clinical trials are currently actively recruiting participants with AD or LBD (clinicaltrials.gov, NCT04571112, NCT02589925, NCT03959124).

This review shows that more preclinical data are needed to design proper future clinical studies. We think that the rather poor clinical effects of NBM DBS so far are related to suboptimal selection of patients and to suboptimal stimulation paradigms. More preclinical evidence is needed to optimize NBM DBS clinically. Animal experiments should focus on the relationship between partially lesioned NBM and the effect of NBM stimulation, in order to guide clinical patient selection. The addition of in vivo cholinergic imaging might contribute to translate the findings into clinical practice, as well as the combination with pharmacological interventions. Last but not least refined preclinical protocols, focusing on the variety of stimulation parameters, should result in recommendations for the clinical setting, whereas electrocortical recordings or deep brain sensing of f.i. local field potentials should be integrated in these protocols.

## Conclusion

In summary, our preclinical systematic review shows the potential benefits of NBM DBS. However, these positive effects should be cautiously interpreted, due to substantial pitfalls in the design, conduct, and reporting of these studies so far. The reporting of the applied NBM stimulation paradigms in animal models should be standardized to increase the comparability and utility.

In our opinion NBM DBS research should focus first on proper preclinical studies, before starting new clinical trials on NBM DBS. We have to justify new trials in this vulnerable target population for NBM DBS, in order to improve the currently rather poor clinical effects of NBM DBS.

## Methods

### Study design

The systematic review of the animal studies was conducted in accordance to methods and guidelines from the Systematic Review Centre for Laboratory Animal Experimentation (SYRCLE)^96,97^. The protocol of the systematic review was registered in the Collaborative Approach to Meta-Analysis and Review of Animal Data from Experimental Studies (CAMARADES) database. Additionally, we reviewed separately all available clinical literature in order to compare the findings in the animal studies.

### Search strategy

Literature search was performed in two electronic databases, MEDLINE and Embase. No time restriction was applied, and all available literature up to November 2020 was reviewed. Two search components were combined: electrical stimulation and the NBM, as described in Supplementary File. Considering that rodent studies often referred the NBM structure to its broader anatomical term, the basal forebrain due to the inherently indistinctive and less-differentiated nature of this structure in rodents than in primates, we therefore included the term “basal forebrain” in our search strategy^77,78^. However, articles which clearly specified the basal forebrain structure as non-NBM, such as the medial septum (Ch1) and the diagonal band of Broca (Ch2 and Ch3), were excluded. Furthermore, we examined articles from the reference lists of the retrieved studies for additional eligible articles.

### Study selection

Two authors (MN and IHP) independently screened the studies retrieved from the database search in two stages. First, all articles were screened based on titles and abstracts if electrical stimulation to the NBM was suggested. Non-original research articles were excluded. Second, full-text screening was performed to identify any data duplicates across studies. Finally, the included articles were classified according to the main outcome of the studies. These outcomes include the behavior, the secretion level of ACh, the effect on the CBF, NGF secretion, and brain glucose metabolism, and the effect on electrophysiological-related neuroplasticity.

### Data extraction

Two authors (MN and IHP) independently extracted the data. The third author (TvL) decided about discrepancies in data extraction between the first two authors. The following data were extracted (1) general information: name of the first author, year of publication; (2) sample characteristics: animal model used, strain, age; (3) stimulation technique: details about the stimulation algorithm, timing and duration, intensity, pulse width, and frequency of stimulation; (4) description of experimental results qualitatively and, for studies with behavioral cognitive outcomes, quantitatively by extracting the mean and standard deviations (SDs) or standard errors (SE). Graphical data were measured using digital ruler software (Plot Digitizer).

### Quality assessment

The quality of animal studies reporting the behavioral outcomes of NBM DBS was independently assessed by two authors (MN and IHP) using the SYRCLE Risk of Bias (RoB) criteria designed for animal studies^96^. The study quality was assessed for its internal validity by examining the risk of possible internal biases including performance bias, exclusion bias, detection bias, and selection bias. In addition, the reporting of electrode positioning verification and the complete reporting of the stimulation parameters were also checked. The RoB was rated high if the assessed methodology clearly introduced bias (see Supplementary Table 1, 2, and 3 for details). The RoB is low if the answer to the rating item was yes, high if the answer was no, or unclear if the explanation in the manuscript was missing.

### Data synthesis

Data of all included studies were analyzed qualitatively and in addition quantitatively for behavioral outcomes. Meta-analysis was performed in animal studies which reported stimulation effects on cognitive tasks and involved comparisons with and without NBM DBS. The meta-analysis was performed using a random effect model by the metafor package in R (version 3.2.2)^98^. Random effect model was used to account for study-level heterogeneity. The standardized mean difference (SMD) of each outcome was calculated by subtracting the mean of the stimulation group to the mean of the sham control divided by the pooled SD of the two groups. SMD of each study was pooled to obtain an overall SMD and 95% confidence interval. Heterogeneity was indicated by the I-squared test. Publication bias was analyzed by Egger’s regression asymmetry test followed by fill-and-trim analysis to correct for publication bias^99^.

## Supplementary Information

Supplementary Information.
