# Supplementary material for: Electrical stimulation of the nucleus basalis of meynert: a systematic review of preclinical and clinical data
Source: Sci Rep. 2021 Jun 3;11:11751. doi: 10.1038/s41598-021-91391-0 (PMC8175342; doi:10.1038/s41598-021-91391-0)
Supplement: Supplementary file 1 — Supplementary Information. [file 41598_2021_91391_MOESM1_ESM.docx]

**SUPPLEMENTARY INFORMATION**

Electrical Stimulation of the Nucleus Basalis of Meynert: A Systematic Review of Preclinical and Clinical Data

Muhammad Nazmuddin^1^, Ingrid H. Philippens^2^, Teus van Laar^1^

*^1^Department of Neurology, Parkinson Expertise Center, University Medical Center Groningen, University of Groningen, Groningen, the Netherlands.*

*^2^Animal Science Department, Biomedical Primate Research Centre (BPRC), P*.O. *Box 3306, 2280 GH, Rijswijk, the Netherlands.*

Correspondence should be addressed to M. N. (email: m.nazmuddin@umcg.nl)

**CONTENT**

1. Search strategy
2. Supplementary figure
3. Supplementary table
4. **Search Strategy**

**PUBMED**

(deep brain stimulation[MeSH Terms] OR electric stimulation[MeSH Terms] OR electric stimulation therapy[MeSH Terms] OR deep brain stimulation*[tiab] OR (electri*[tiab] AND stimul*[tiab]) OR electrotherapy[tiab] OR electrostimul*[tiab])

AND

basal nucleus of Meynert[MeSH Terms] OR basal nucleus[tiab] OR Meynert[tiab] OR nucleus basalis[tiab] OR Basal nucleus[tiab] OR (Nucleus[tiab] AND magnocellular*[tiab]) OR Nucleus basalis magnocellularis[tiab] OR Meynert basal nucleus[tiab] OR nucleus basalis of Meynert[tiab] OR (Basal*[tiab] AND magnocellular*[tiab] AND Nucleus[tiab]) OR basal forebrain[tiab]

Hits: 787

**Embase**

(‘brain depth stimulation’)/de OR (‘electrostimulation’)/de OR (deep brain stimulation*):ti,ab OR (electri*AND stimul*):ti,ab OR (electrotherapy):ti,ab OR (electrostimul*):ti,ab

(’nucleus basalis magnocellularis’)/de OR (’Meynert basal nucleus’)/de OR (basal AND nucleus AND Meynert):ti,ab OR (nucleus basalis):ti,ab OR (Nucle* AND magnocellular*):ti,ab OR (nucleus basalis magnocellularis):ti,ab OR (Meynert basal nucleus):ti,ab OR (nucleus basalis Meynert):ti,ab OR (basal* AND magnocellular* AND nucle*):ti,ab OR (nucleus AND Meynert):ti,ab OR (basal forebrain):ti,ab

Hits: 567

Total hits without exclusion: 1254

duplicates removed: 186

Total articles after removing duplicates: 1068

1. **Supplementary figure**

**
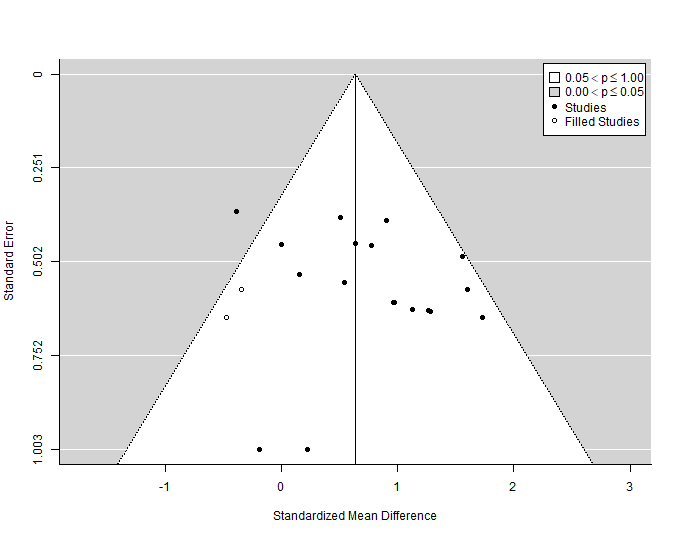
**

**Supp. Fig. 1.** Funnel plots for behavioural cognitive performance showed the outcome distribution of the sham-controlled and the repetitive-measure studies to estimate potential publication bias.

1. **Supplementary table**

**Supplementary table 1.** The summary of risk of bias assessment in sham-controlled studies reporting the behavioural cognitive performance effect of NBM-ES

| **No** | **Aspect of bias** | **Cue Question** | **I. Sham vs NBM Stimulation (Rodent study)** | | | | | |
| --- | --- | --- | --- | --- | --- | --- | --- | --- |
|  |  |  | **Montero-Poster 2001** | **Montero-Poster 2004** | **Boix-Trelis et al 2006** | **Reed et al 2011** | **Lee et al 2016** | **Huang et al 2019** |
| **1** | **Randomisation** | Was the allocation sequence adequately generated and applied? | unclear | unclear | unclear | unclear | unclear | unclear |
| **2** | **Similar baseline** | Were the groups similar at baseline or were they adjusted for confounders in the analysis? | yes | yes | yes | yes | yes | yes |
| **3** | **Selection blinding** | Was the allocation to the different groups adequately concealed? | unclear | unclear | unclear | unclear | unclear | unclear |
| **4** | **Random housing** | Were the animals randomly housed during the experiment? | unclear | unclear | unclear | unclear | unclear | unclear |
| **5** | **Investigation blinding** | Were the caregivers and investigators blinded from knowledge which intervention each animal received during the experiment/session? | unclear | unclear | unclear | unclear | unclear | unclear |
| **6** | **Random assessment** | Were animals selected at random for outcome assessment? | unclear | unclear | unclear | yes | unclear | unclear |
| **7** | **Assessor blinding** | Was the outcome assessor blinded? | unclear | unclear | unclear | yes | yes | yes |
| **8** | **Data attrition** | Were incomplete outcome data adequately addressed? | yes | yes | yes | yes | yes | yes |
| **9** | **Incomplete reporting** | Are reports of the study free of selective outcome reporting? (*) | yes | yes | yes | yes | yes | yes |
| **10** | **Other bias** | Was the study apparently free of other problems that could result in high risk of bias? (*) | yes | yes | yes | yes | yes | yes |
|  | | *Was the study free of contamination (pooling drugs)? | yes | yes | yes | yes | yes | yes |
|  |  | *Was the study free of inappropriate influence of funders? | yes | yes | yes | yes | yes | yes |
|  |  | *Was the study free of unit of analysis errors? | yes | yes | yes | yes | yes | yes |
|  |  | *Were design-specific risks of bias absent? | yes | yes | yes | yes | yes | yes |
|  |  | *Were new animals added to the control and experimental groups to replace drop-outs from the original population? | yes | yes | yes | yes | yes | yes |
| **11** | **Stimulation parameters** | NBM DBS stimulation parameters mentioned? Pattern, shape, frequency, pulse width, amplitude | unclear | unclear | unclear | yes | yes | unclear |
| **12** | **Electrode verification** | Electrode placement verified by intra-operative neurophysiological recording or by post-mortem assessment? | yes | yes | yes | no | yes | no |

**Supplementary table 2.** The summary of risk of bias assessment in studies reporting the behavioural cognitive performance effect of NBM-ES when paired versus unpaired with cue presentation

| **No** | **Aspect of bias** | **Cue question** | **II. Unsynchronized vs Synchronized Stimulation (Rodent study)** | | | | | |
| --- | --- | --- | --- | --- | --- | --- | --- | --- |
|  |  |  | **McLin et al 2002** | **Weinberger et al 2006** | **Miasnikov et al 2006** | **Miasnikov et al 2009** | **Weinberger et al 2009** | **Miasnikov et al 2011** |
| **1** | **Randomisation** | Was the allocation sequence adequately generated and applied? | unclear | unclear | unclear | unclear | unclear | unclear |
| **2** | **Similar baseline** | Were the groups similar at baseline or were they adjusted for confounders in the analysis? | yes | yes | yes | yes | yes | yes |
| **3** | **Selection blinding** | Was the allocation to the different groups adequately concealed? | unclear | unclear | unclear | unclear | unclear | unclear |
| **4** | **Random housing** | Were the animals randomly housed during the experiment? | unclear | unclear | unclear | unclear | unclear | unclear |
| **5** | **Investigation blinding** | Were the caregivers and investigators blinded from knowledge which intervention each animalr eceived during the experiment/session? | unclear | unclear | unclear | unclear | unclear | unclear |
| **6** | **Random assessment** | Were animals selected at random for outcome assessment? | unclear | unclear | unclear | unclear | unclear | unclear |
| **7** | **Assessor blinding** | Was the outcome assessor blinded? | unclear | unclear | unclear | unclear | unclear | unclear |
| **8** | **Data attrition** | Were incomplete outcome data adequately addressed? | yes | yes | yes | yes | yes | yes |
| **9** | **Incomplete reporting** | Are reports of the study free of selective outcome reporting? (*) | yes | yes | yes | yes | yes | yes |
| **10** | **Other bias** | Was the study apparently free of other problems that could result in high risk of bias? (*) | yes | yes | yes | yes | yes | yes |
|  | | *Was the study free of contamination (pooling drugs)? | yes | yes | yes | yes | yes | yes |
|  |  | *Was the study free of inappropriate influence of funders? | yes | yes | yes | yes | yes | yes |
|  |  | *Was the study free of unit of analysis errors? | yes | yes | yes | yes | yes | yes |
|  |  | *Were design-specific risks of bias absent? | yes | yes | yes | yes | yes | yes |
|  |  | *Were new animals added to the control and experimental groups to replace drop-outs from the original population? | yes | yes | yes | yes | yes | yes |
| **11** | **Stimulation parameters** | NBM DBS stimulation parameters mentioned? Pattern, shape, frequency, pulse width, amplitude | no | yes | yes | yes | yes | yes |
| **12** | **Electrode verification** | Electrode placement verified by intra-operative neurophysiological recording or by post-mortem assessment? | yes | yes | yes | yes | yes | yes |

**Supplementary table 3.** The summary of risk of bias assessment in studies reporting the behavioural cognitive performance effect of NBM-ES with repetitive-measure design

| **No** | **Aspect of bias** | **Cue question** | **III. Single-group, repeated-measures study with non-human primates & rodents** | | | | | | |
| --- | --- | --- | --- | --- | --- | --- | --- | --- | --- |
|  |  |  | **Miasnikov et al 2008a** | **Miasnikov et al 2008b** | **Avila & Lin 2014** | **Mayse et al 2015** | **Liu et al 2017** | **Liu et al 2018** | **Koulousakis et al 2019** |
| **1** | **Randomisation** | Was the allocation sequence adequately generated and applied? | n.a. | n.a. | n.a. | n.a. | n.a. | n.a. | n.a. |
| **2** | **Similar baseline** | Were the groups similar at baseline or were they adjusted for confounders in the analysis? | n.a. | n.a. | n.a. | n.a. | n.a. | n.a. | n.a. |
| **3** | **Selection blinding** | Was the allocation to the different groups adequately concealed? | n.a. | n.a. | n.a. | n.a. | n.a. | n.a. | n.a. |
| **4** | **Random housing** | Were the animals randomly housed during the experiment? | unclear | unclear | unclear | unclear | unclear | unclear | unclear |
| **5** | **Investigation blinding** | Were the caregivers and investigators blinded from knowledge which intervention each animal received during the experiment/session? | no | no | no | no | no | no | no |
| **6** | **Random assessment** | Were animals selected at random for outcome assessment? | unclear | unclear | unclear | unclear | unclear | unclear | yes |
| **7** | **Assessor blinding** | Was the outcome assessor blinded? | unclear | unclear | unclear | unclear | unclear | unclear | unclear |
| **8** | **Data attrition** | Were incomplete outcome data adequately addressed? | yes | yes | yes | yes | yes | yes | yes |
| **9** | **Incomplete reporting** | Are reports of the study free of selective outcome reporting? | yes | yes | yes | yes | yes | yes | yes |
| **10** | **Other bias** | Was the study apparently free of other problems that could result in high risk of bias? (*) | no | no | no | no | no | no | no |
|  | | *Was the study free of contamination (pooling drugs)? | yes | yes | yes | yes | yes | yes | yes |
|  |  | *Was the study free of inappropriate influence of funders? | yes | yes | yes | yes | yes | yes | yes |
|  |  | *Was the study free of unit of analysis errors? | yes | yes | unclear | unclear | yes | yes | yes |
|  |  | *Were design-specific risks of bias absent? | no | no | no | no | no | no | no |
|  |  | *Were new animals added to the control and experimental groups to replace drop-outs from the original population? | yes | yes | yes | yes | yes | yes | yes |
| **11** | **Stimulation parameters** | NBM DBS stimulation parameters mentioned? Pattern, shape, frequency, pulse width, amplitude | yes | yes | yes | yes | yes | yes | yes |
| **12** | **Electrode verification** | Electrode placement verified by intra-operative neurophysiological recording or by post-mortem assessment? | yes | yes | yes | yes | yes | yes | yes |

**Supplementary table 4.** Characteristics of animal studies with information on the effect of NBM DBS on cortical acetylcholine release.

| **No.** | **Author, year** | **Sex** | **Species, Strain, Sex** | **Sample size** | **Age** | **Weight** | **Consciousness State** | **Experimental Design** | **Bilateral / Unilateral** | **Stimulation parameter** | **Cortical Regions Observed** | **Measuring Technique** | **Duration of Stimulation Session** | **Results** |
| --- | --- | --- | --- | --- | --- | --- | --- | --- | --- | --- | --- | --- | --- | --- |
| 1 | Casamenti et al, 1986 ^1^ | male | Wistar rats | 5 | NA | 150-180 gr | Freely moving rats | longitudinal observations every 20 mins, starting 40 mins before stimulation up to 60 mins after the start of stimulation | Unilateral | 30 Hz; 250 μs; 100 μA; intermittent: 1-s on/3-s off | Ipsilateral Fronto-parietal cortex | Cortical cups technique | 20 minutes | Electrical stimulation of the NBM significantly induce cortical Ach release during 20 minutes of stimulation and the 20-min period after the start of the stimulation. |
| 2 | Kurosawa et al, 1989 ^2^ | NA | Fischer-344 rats | 6 (adult group); 6 (aged group) | 6-8 months (adult group); 27-28 months (aged group) | NA | Under halothane anesthesia | longitudinal observations, sample at the prestimulus stage was compared to sample after 10-min stimulation. | Unilateral | 50 Hz; 50, 100, 200, 500 μA; 500 μs | Ipsilateral parietal cortex | Microdialysis technique | 10 minutes | no different stimulation response between aged and healthy adult group |
| 3 | Kurosawa et al, 1989 ^3^ | NA | Fischer-344 rats | 6 (stimulation amplitude), 6 (stimulation frequency) | NA | 350-440 gr | Under halothane anesthesia | longitudinal observations every 10 mins, starting 30 mins before stimulation up to 60 mins after the start of stimulation | Unilateral | 0, 1,2, 5, 10, 20, 50, 100 Hz; 0, 20, 50, 100, 200, 500 μA; 500 μs | Ipsilateral parietal cortex | Microdialysis technique | 10 minutes | electrical stimulation of the NBM induced significant ACh release in the parietal cortex with the peak increase at 20 Hz while the increase of Ach was along the increase of the stimulation intensity up to 500 μA |
| 4 | Rasmusson et al, 1992 ^4^ | female | Sprague-Dawley rats | 43, 17 were excluded from the analysis | NA | 200-300 gr | Under urethane anesthesia | longitudinal observations every 20 mins, starting 40 mins before stimulation period 1, up to 40 minutes after the end of stimulation period 2 | Unilateral | 10 x 1000-μs burst stimulation of 2000 μA. Each burst deliver electrical pulses in varied frequency of 10, 50, 100, and 200 Hz | somatosensory cortex | Microdialysis technique | 20 minutes | 3-4 fold increase in Ach release by 100 Hz stimulation; The increase at this frequency was two times higher than the Ach release at lower and higher frequency |

**Supplementary table 5.** Characteristics of animal studies with information on the effect of NBM DBS on cerebral blood flow, metabolism, and neural growth factor (NGF) secretion

| **No.** | **Author, year** | **Species, strain, sex** | **Sample**  **size** | **Age** | **Weight** | **States** | **Comparisons** | **Bilateral / Unilateral (Right/left)** | **Stimulation parameter** | **Brain**  **regions** | **Technique** | **Stimulation Duration** | **Main Results** |
| --- | --- | --- | --- | --- | --- | --- | --- | --- | --- | --- | --- | --- | --- |
|  |  |  |  |  |  |  |  |  |  |  |  |  |  |
| 1 | Kurosawa et al, 1989 ^3^ | rats, Fischer-344 | 6 (adult); 6 (aged) | 6-8 months (adult group); 27-28 months (aged group) | NA | Under halothane anesthesia | longitudinal observations, control sample at the prestimulus stage was compared to sample after 10-min stimulation. Response between healthy adult vs aged rats was observed | Unilateral | 50 Hz; 50, 100, 200, 500 µA; 500 µs | Ipsilateral parietal cortex | laser Doppler flowmetry | 10 minutes | No significant difference of the stimulation response on the CBF between adult and aged rats |
| 2 | Biesold et al, 1989b ^5^ | rats, Wistar | 24 | NA | 305-370 g | under urethane anesthesia | comparison of CBF across different stimulus intensity | Unilateral, left or right NBM | 50 Hz; 10, 20, 50, 100, 200, 500, 1000 µA; 0.5 ms | Ipsilateral parietal cortex | laser Doppler flowmetry | 10-20 s | significant increase of CBF starting from 50 µA stimulation |
| 3 | Adachi et al, 1990 ^6^ | rats, Wistar, male | 25 | Adult | 280-420 g | under urethane anesthesia | comparisons of CBF response across frontal, parietal, and occipital cortices, and comparisons between pre- and post-stimulus states. The magnitude of the peak response was measured. | Unilateral, left or right NBM | 50 Hz; 200 µA; 500 µs | Ipsilateral and contralateral frontal, parietal, and occipital cortex | laser Doppler flowmetry | 10 s | NBM-ES enhanced CBF significantly in all three cortices ipsilateral to the stimulation site. The response of stimulation in the NBM was significantly larger in the frontal cortex than in the parietal and occipital cortices |
| 4 | Hallstrom et al, 1990 ^7^ | rats, Wistar, male | 6 | NA | 290-310 g | Under halothane anesthesia | longitudinal observations 10 mins pre-, during, and post-stimulation | Unilateral | 50 Hz; 200 µA; 500 µs | Ipsilateral parietal cortex | laser Doppler flowmetry | 10 minutes | NBM-ES enhanced cortical CBF, independent of changes in extracellular lactate or in systemic arterial blood pressure |
| 5 | Adachi et al, 1990 ^8^ | rats, Wistar | 24 | NA | 260-380 g | Under halothane anesthesia | group comparisons: NBM-not-stimulated group, NBM-stimulated group, MS-not-stimulated sham group, MS-stimulated group | Unilateral | 50 Hz; 200 µA; 500 µs | olfactory bulb, frontal cortex, hindlimb area of parietal cortex (HL), somatosensory area of parietal cortex (S1), occipital cortex, caudate putamen, hippocampus, thalamus + hypothalamus, superior colliculus, inferior colliculus, midbrain, cerebellum, pons and medulla | [14C]iodoantipyrine autoradiography | 60 s | Enhanced CBF in the frontal, parietal and occipital cortices ipsilateral to the stimulated NBM while the stimulation of the unilateral MS produced significant increases in bilateral hippocampal blood flow. |
| 6 | Kimura et al 1990 ^9^ | Rats, Wistar, male | 15 (n sham=6; stim=6; CBF assessment group) | adult | 240-350 g | Under halothane anesthesia | glucose metabolism between NBM stimulation vs no stimulation | Unilateral | 200 µA, 500 us, 50 Hz | cerebral cortex, basal ganglia, limbic structures, diencephalon, brain stem, cerebellum | [14C]2-deoxyglucose autoradiography , laser Doppler flowmetry | 45 minutes | NBM-ES increased cortical CBF without affecting metabolisms. |
| 7 | Sato & Sato 1990 ^10^ | rats | NA | NA | NA | under urethane or halothane anesthesia | longitudinal observation, pre- and post-stimulation | Unilateral | 50 Hz, 500 us, 10-1000 µA | somatosensory cortex | laser Doppler flowmetry, microdyalisis | 10 s | NBM-ES enhanced CBF and ACh release. The vasodilation effect can be abolished by applying muscarinic and nicotinic receptor antagonist |
| 8 | Linville & Arneric 1991 ^11^ | rats, Sprague-Dawley, male | 7 (young); 6 (aged) | 4-6 months, 22-26 months | NA | under chloralose anesthesia | longitudinal observation | Unilateral | 2.5, 25, 50, 100, 150 Hz; 2000 us; 25, 50, 75, 100, and 150 µA | cortical and subcortical area | 14C-IAP autoradiography, laser Doppler flowmetry | 10 s | Ageing decreased CBF enhancement response elicited by basal forebrain stimulation |
| 9 | Adachi et al, 1992 ^12^ | Wistar rats | 6 | adult | 300-450 g | under urethane anesthesia | longitudinal observations pre- and post-stimulation states were measured | Unilateral | 50 Hz; 200 µA; 500 µs | parietal cortex | video microscope (diameter of pial artery), laser Doppler flowmetry (CBF) | 1 min | The increase in cortical CBF elicited by NBM stimulation was independent from the diameter of pial arteries |
| 10 | Adachi et al, 1992 ^13^ | Wistar rats | 9 | adult | 290-400 g. | under urethane anesthesia | series of longitudinal experiments in the same animals | Unilateral | 50 Hz; 200 µA; 500 µs | parietal cortex | laser Doppler flowmetry | 10 s | NO involved as a cofactor of vasodilation following NBM-ES |
| 11 | Raszkiewicz et al 1992 ^14^ | rats, Sprague-Dawley, male | L-arginine-treated (N = 4), L-NNA-treated (N = 6), D-NNA-treated (N = 3) | NA | NA | under urethane anesthesia | longitudinal observation | Unilateral | cathodal pulse, 10-s trains, 50 Hz, 2000 us, 100 µA | frontal cortex | laser Doppler flowmetry | 10 s | NO synthase mediated the cortical vasodilation elicited by NBM-ES |
| 12 | Linville et al 1992 ^15^ | rats, Sprague-Dawley, male | untreated H-PHY (n=6), H-PHY -treated (n=6) | 2-4 months | NA | under urethane anesthesia | longitudinal observation | Unilateral | cathodic pulse, 5, 10, 25, and 50 Hz, 100 µA | frontoparietal cortex | laser Doppler flowmetry | NA | heptyl-physostigmine, a cholinesterase inhibitor, enhanced the corticovasodilation elicited by BF stimulation |
| 13 | Linville et al 1993 ^16^ | rats, Sprague-Dawley, male | 6 | NA | 300-400 g | under halothane anesthesia | cortically-intact vs cortically-lesioned hemisphere, longitudinal observation | Bilateral | cathodic pulse, 10 s trains of 2 ms duration pulses at a frequency of 5, 10, 25, 50 Hz and intensity of 100 µA | frontal cortex | laser Doppler flowmetry | 10 s | NBM-stimulation-induced cortical vasodilation response is not different between the cortically-intact hemisphere and the cortically-lesioned hemisphere |
| 14 | Linville et al 1993 ^17^ | rats, Sprague-Dawley, male | 7 (-)-nicotine; 5 (+)-nicotine; 6 (-)-Iobeline; 4 (-)-cystisine | 2-4 months | NA | under urethane anesthesia | longitudinal observation, comparisons between pharmacological treatment | NA | cathodic pulse, 10 s trains of 2 ms duration pulses at a frequency of 5, 10, 25, 50 Hz and intensity of 100 µA | frontal cortex | laser Doppler flowmetry | 10 s | cortical CBF may be enhanced by NBM-ES. Some structural classes of nicotinic agonists boost the vasodilation response elicited by the BF activation |
| 15 | Sercombe et al 1994 ^18^ | rats, Fischer 344, male | 13 (young group); 10 (aged group) | 2-4 months | 300 g | 2 hrs after anesthesia ended | longitudinal observation before and after physostigmine treatment, comparisons between young and aged group | Bilateral | intermittent 1s ON/OFF, cathodic pulse, 200 Hz, 500 us, 50 µA | frontal and parietal cortex | helium clearence (CBF), mass spectrometry (tissue partial pressure) | 1.5-2.5 mins | ageing decreases CBF enhancement response elicited by basal forebrain activation. The efficacy of physostigmine to boost the CBF enhancement decreases by ageing |
| 16 | Vaucher et al, 1995 ^19^ | Rats, Sprague-Dawley, male | 14 | NA | NA | conscious rats | interhemispheric difference and stimulation vs sham control group | Unilateral, right | 100 Hz; 50 µA; 500 µs; cyclic 1 s ON/1 s OFF for autoradiographic CBF measurement; 10 s continuous stimulation for exploratory experiment using LDF | cortical and subcortical structures | [14C]iodoantipyrine autoradiography & laser Doppler flowmetry | 20 mins | CBF increases in most neocortical areas (more than four times fold in the frontal area) and some neocortical areas including zona incerta, parts of thalamic nuclei, and extrapyramidal structures. |
| 17 | Zhang et al 1995 ^20^ | rat, Sprague-Dawley, male | 5 (atropine-treated); 6 (mecamylamine-atropine-treated); 8 (vehicle-treated); 6 (NOS-inhibitor-treated); | NA | 290-380 g | under halothane anesthesia | longitudinal observation (before and after treatment), comparison across pharmacological treatment | unilateral | cathodic, 50 Hz, 500 us, 100 µA | parietal cortex | laser Doppler flowmetry, nitric oxide synthase assay | 8 s | NBM-Es leads to local cortical release of ACh which in turn activates endothelial NOS and results in vasodilation responses |
| 18 | Iadecola & Zhang 1996 ^21^ | rat, Sprague-Dawley, male | 56 | NA | 290-380 g | under halothane anesthesia | longitudinal observation (before and after treatment), comparison across pharmacological treatment | Unilateral | cathodic, 50 Hz, 500 us, 100 µA | frontoparietal cortex | laser Doppler flowmetry, nitric oxide synthase assay | 8 s | NO is the major mediator of smooth muscle relaxation in the NBM-ES-induced vasodilation |
| 19 | Lacombe et al, 1997 ^22^ | Rats | NA | 2-4 mo (young adult group); 22-28 mo (aged group) | NA | conscious rats | young vs aged rats | Unilateral | 50 µA, 100 Hz, 500 µs, intermittent 1 s on/ 1 s off stimulation | frontal, parietal crotices | mass spectrometry (helium clearance), quantitative radiography: [14C]iodoantipyrine for CBF measure and [14C]deoxyglucose to measure glucose utilization | NA | electrical stimulation of the NBM significantly enhanced CBF of the frontral and parietal cortex. The increase was more dominant in the frontal cortex compared to the parietal cortex. The CBF increase after electrical stimulation of the NBM was twice higher in young compared to aged rats. The CBF increase was uncoupled with the increase of the glucose utilization value in the corresponding brain region. |
| 20 | Vaucher et al, 1997 ^23^ | Rats, Sprague-Dawley, male | 13 | NA | 300-320 g. | conscious rats | interhemispheric difference and stimulation vs sham control group | Unilateral, right | 100 Hz; 50 µA; 500 µs; cyclic 1 s ON/OFF | cortical and subcortical structures | [14C]2-deoxyglucose autoradiography | 5 mins | the regulation of the cerebral circulation at the cortical and subcortical level is not coupled with changes in its local metabolic activity |
| 21 | Uchida et al, 2000 ^24^ | Rats, male and female | 35 | young adult (4±7 months), old (24±25 months), and very old (32±42 months) rats | NA | under urethane anesthesia | series of longitudinal experiments in the same animals | Unilateral | (0.5 ms, 20-200 mA, 50 Hz) for 1.5 min | frontal and parietal cortex | laser Doppler flowmetry | 1.5 min | The vasodilation responses in frontal and parietal cortices by NBM-ES were similar in young and old rats, but declined significantly in very old rats while stimulation-induced release of ACh was similar across ages. No differences in the CBF changes elicited by administration of a muscarinic-receptor agonist across ages. |
| 22 | Hotta et al, 2002 ^25^ | Rats, male | 45 (n CBF exp= 19; n hist exp=26) | adult, age | 300-410 g. | Under halothane anesthesia | CBF change between NBM stimulation vs no stimulation | Unilateral, left | rectangular pulse current; 50 Hz; 200 µA; 500 µs; cyclic 1 s ON/OFF | frontal, parietal, occipital cortex | laser Doppler flowmetry | 65 minutes, starting 5 minutes before the intermittent occlusion | NBM-ES prevented the occlusion-induced CBF decrease in all three cortices and reduced delayed neuronal death in the cortices due to artificial vascular occlusion |
| 23 | Hotta et al, 2004 ^26^ | Rats, Wistar, male | 8 (n control=4; n stim=4) | 5 mo | 330-380 g. | under urethane anesthesia | CBF change and the mean inner diameter between NBM-ES vs no stimulation | Unilateral | 200 μA, 0.5 ms, 50 Hz, 1 s on/1 s off | parietal cortex | laser Doppler flowmetry, histology | 11.5-15 min; 1.5-5 min before the start of tissue fixation | NBM-ES enlarged the inner diameter of the parenchymal blood vessels |
| 24 | Seigneur et al 2006  ^27^ | cat, male & female | adult | NA | NA | under anesthesia and awake state | CBF change in association with neuronal and glial cellular electrophysiological activity | Unilateral | 1–3 trains of 70 pulses at 100 Hz, 100 µA | suprasylvian area | extracellular K+ recording, local field potential, laser Doppler flowmetry | periodic, duriing experiment | NBM-ES hyperpolarized >80% glial cells, depolarized neurons, decreased extracellular K+ concentration, increased membrane resistance, increased CBF, decreased membrane capacitance, and persistent positive DC field potentials |
| 25 | Hotta et al 2007 ^28^ | rat, Wistar, male | 10 | 4-5 months | 340-420 g | under halothane anesthesia | longitudinal observation before and after NBM-ES | unilateral | 200 μA, 0.5 ms, 50 Hz, 1 s on/2 s off | parietal cortex | microdialysis-ELISA, laser Doppler flowmetry | 100 minutes | NGF was increased significantly in the ipsilateral cortex during 200–500 min after the end of NBM-ES |
| 26 | Hotta et al 2007b ^29^ | cat, male & female | 7 | NA | NA | under halothane anesthesia | longitudinal observation before and after NBM-ES | unilateral | 20 Hz, 500 us, 0.1–5 mA for 20 s | somatosensory cortex | laser Doppler flowmetry | during experiment | NBM-ES enhanced CBF in the ipsilateral primary somatosensory cortex |
| 27 | Kocharyan et al 2007 ^30^ | rat, Sprague-Dawley, male | NA | NA | 280-320 g | under urethane anesthesia | longitudinal observation before and after NBM-ES + pharmacological treatment | unilateral, left | 100 Hz, 50 μA, 0.5 ms, 1 s on/1 s off | frontal cortex | laser Doppler flowmetry, cFOS immunohistochemistry | 5 minutes | the cortical vasodilation response of NBM-ES was facilitated by the cholinergic activation of layers II to VI SOM- and/or NPY-containing interneurons, as well as layer I GABA interneurons |
| 28 | Hotta et al 2009 ^31^ | Rats, Wistar, male | 36 (n adult=31; n aged=5) | adult 4-6 months, aged 29-31 months | 300-450 g | Under halothane anesthesia | NGF secretion induced by NBM-ES in adult vs aged rats | Unilateral NBM, left or right | Intermittent, 1s on/2s off, biphasic, 50 Hz, 500 us, 200 µA | parietal cortex | microdialisis-ELISA, immunohistochemistry | 100 minutes | NBM-ES induced NGF secretion via nicotinic receptor up to 500 minutes post-stimulation in adult, but not in aged rats. |
| 29 | Hotta et al, 2011 ^32^ | C57BL/6NCr mice | 18 | NA | 30-40 g. | under urethane anesthesia | CBF change across longitudinal observation | Unilateral, left | 50 Hz, 50 μA, 500 ms | frontal, parietal, occipital cortex | laser speckle (whole dorsal surface of the brain, n=7), laser Doppler flowmetry (n=9; parietal cortex) | 10 s | Increased CBF in ipsilateral frontal, parietal and occipital cortices by NBM-ES is independent of changes in systemic blood pressure. CBF changes by stimulation at lower amplitude (two times amplitude threshold) involved muscarinic-dependent activation while stimulation at three times stimulation amplitude possibly recruited activation of nicotinic-gated pathway in addition to muscarinic activation. The response on CBF is associated with the number of cholinergic neurons surrounded by the electrode tip. |
| 30 | Uchida et al, 2011 ^33^ | Rats, Wistar, male | 23 | 4-9 mo | 320-430 | under urethane anesthesia | CBF change during NBM-ES between animals with subcutaneous nicotine vs saline injection | Unilateral NBM, left or right | 17/50 Hz, 10-200 µA, 500 µs | parietal cortex | laser Doppler flowmetry | 90 s/3mins | The threshold amplitude of NBM-ES to increase ACh level and CBF decreased significantly in nicotine-treated rats (20 μA) compared to control rats (50 μA). |
| 31 | Takata et al, 2013 ^34^ | Mice, C57BL/6J | for Ca elevation: Wild type (n=3) vs KO (n=3) | >9 mo | NA | under urethane anesthesia | CBF change compared to pre-stimulus. Qualitative comparison of the CBF pattern between the IP3R2 strain vs C57BL/6J strain | Unilateral | 100 Hz, 0.5 ms, 50/200 µA | primary somatosensory cortex | laser Doppler flowmetry | single train of 50 pulses | Both whisker tactile stimulation and NBM-ES induced CBF changes in similar degrees for both IP3R2-KO mice and WT mice. At 200 µA NBM-ES, the CBF increased immediately and followed by a transient overshot decrease both in WT and KO mice. |
| 32 | Hotta et al, 2013 ^35^ | Mice, C57BL/6NCr, male | 7 | 6 mo | 22-38 g | under pentobarbital-isoflurane anesthesia | the diameter of single penetrating arteries | Unilateral, left | 0.5 ms, 30-50 μA, 50 Hz, 1-s on/1-s off | frontal cortex | two-photon microscopy | 5-10 minutes | NBM-ES increased the diameter of penetrating arteries penetrating through different cortical layers by ± 10%. The enlargement began within 1 second after the onset of NBM-ES in the upper cortical layers, and later in lower layers. |

**Supplementary table 6.** Characteristics of animal studies with information on the effect of NBM DBS on cortical-subcortical plasticity and connectivity with the NBM

| **No.** | **Author, year** | **Species, strain, sex** | **Age** | **Weight** | **States** | **Bilateral / Unilateral Stimulation** | **Stimulation parameter** | **Brain Regions** | **Technique** | **Stimulation Duration** | **Main Results** |
| --- | --- | --- | --- | --- | --- | --- | --- | --- | --- | --- | --- |
|  |  |  |  |  |  |  |  |  |  |  |  |
| **I. Studies related to auditory system** | | | | | | | | | | | |
| 1 | Koch & Ebert 1992 ^36^ | rat, Wistar, male | adult | 200-300 g | under chloral hydrate anesthesia, and awake state | unilateral | 50 us, 5 V | the caudal pontine reticular nucleus (PnC) | extracellular recording | electrical stimuli were paired 5 ms prior to tone stimuli | electrical stimulation of the caudal NBM facilitates the tone-evoked response of th PnC neurons |
| 2 | Metherate & Ashe 1993 ^37^ | Sprague Dawley Rats, male | NA | 250-350 g | Under urethane anesthesia | unilateral, right | single burst of 20-100 pulses at 200 Hz, 100 us pulse width, 100-500 uA amplitude | Auditory cortex | in vivo whole cell recording | single burst stimulation, the effects was observed for and dissipated after 5-10 s. | Electrical stimulation of the NBM eliminates long-lasting, spontaneous hyperpolarization of cortical neuron by muscarinic blocakde of spontaneous K+ current and increase the frequency of all-or-none depolarization event. |
| 3 | Metherate & Ashe 1993 ^38^ | Rats, Sprague-Dawley, male | adult | 250-350 g | Under urethane anesthesia | unilateral. | single burst of 100-500 ms at 10, 40, 100, 200 Hz, 100 µs pulse width, 100-500 uA | Auditory cortex | evoked field potential and unit activity recording, EEG, intracellular recording | immediate effect post-burst stimulation | Electrical stimulation of the NBM potentiates responses of auditory cortex to medial-geniculate thalamic input and the effects differs depending on the stimulation frequency and the duration of the burst. |
| 4 | Hars et al 1993 ^39^ | rat, Wistar, male | adult | 290-390 g | awake state | unilateral | 100 Hz, 100 µs, 80-250 uA | auditory cortex | single unit recording | 300 ms stimulation paired with tone stimuli for 20 times | Pairing electrical NBM stimulation with auditory stimuli enhances the tone-evoked response in the auditory cortex up to 15 minutes after the pairing which is abolished by the administration of atropine |
| 5 | Edeline et al 1994 ^40^ | rat, Wistar, male | adult | 290-390 g | Under urethane anesthesia | unilateral | 300ms train, 100 Hz, 100 µs at low (minimum 20 µA) to high (minimum 100 µA) amplitude | auditory, frontal, and parietal cortices | single unit recording | electrical stimuli were paired 50 ms prior to tone stimuli | NBM electrical stimulation facilitates auditory evoked responses in the auditory cortex. The duration of facilitation depends on the intensity of the stimulation |
| 6 | Bakin & Weinberger 1996 ^41^ | rat, Sprague-Dawley, male | 2.5-4.5 months | 366-516 g | Under urethane anesthesia | unilateral | biphasic, 500 ms train, 200 Hz, 200 µs, 270 uA | auditory cortex | single unit recording | electrical stimuli either paired or unpaired with tone stimuli | Paired tone-NBM stimulation, but not unpaired stimulation, induced auditory receptive field plasticity |
| 7 | Brett & Barth 1997 ^42^ | rat, Sprague-Dawley | NA | 300-500 g | under anesthesia | unilateral, right | 500 Hz, 500 us, 15 µA, 500-ms train | auditory cortex | evoked field potential | periodically during recording experiment | NBM stimulation did not evoke gamma oscillation in the auditory cortex |
| 8 | Kilgard & Merzenich 1998 ^43^ | Sprague Dawley Rats, male | NA | 300 g | awake state | unilateral, right | biphasic pulses, 100 Hz, 100 us pulse width, 70-150 µA | Auditory cortex | electrophysiological mapping with microelectrode recording | 300 trials per day for 20-25 days, effects observed the day after the training | Pairing electrical NBM stimulation with a pure tone in a specific frequency generates broaden receptive field representation of the tone frequency in the auditory cortex |
| 9 | Bjordahl et al 1998 ^44^ | Guinea Pig | adult | 342-504 g | awake | unilateral, left | 100-400 µA, monophasic, 0.2-ms square wave, 200 Hz, total train duration = 500 ms | Auditory cortex | unit cluster recording | 1-day trial, 40 pairing trials, RF plasticity observerd immediately within 1 hour post-stimulation but dissipated 24 hr after stimulation | Animals of which NBM stimulation effectively induced cortical desynchronization develop conditioned-specific receptive-field plasticity after pairing the conditioned tone with electrical stimulation of the NBM |
| 10 | Kilgard & Merzenich 1998b ^45^ | rat, female | NA | 300 g | awake state | unilateral | biphasic, 100 Hz, 100 us, 70-150 µA | auditory cortex | single unit recording | 200 ms NB stimulation train is paired with auditory stimulation, 300 times for 25 days | The response of auditory cortex (the spike rate) after pairing NBM electrical stimulation to tone stimuli depends on the feature of the tone, specifically, the number of tone presentation per second |
| 11 | Dimyan & Weinberger 1999 ^46^ | Guinea Pig, male | adult | 343-756 g | under nembutal anesthesia | unilateral | 500-ms train, biphasic, 200 Hz, 200 us, 50-500 µA | auditory cortex | multi-unit activity recording | tone stimuli paired with NBM stimulation | learning-induced RF plasticity is assisted by basal forebrain stimulation and is experience-dependent |
| 12 | Miasnikov et al 2001 ^47^ | Rats, Sprague-Dawley, male | NA | 413+/-78 g | under urethane anesthesia | Unilateral, right | 100 Hz, 200 us, 200-500 µA, 100-600 ms | Auditory cortex | single unit recording | 30 trials of pairing NBM stimulation & tone, effect was observed 40-170 times post-conditioning | Paired CS-ES of the NBM elicits increased firing rates of neurons to the CS-frequency tone and is prevented by applying muscarinic antagonist to the auditory cortex |
| 13 | Cruikshank & Weinberger, 2001 ^48^ | Sprague Dawley Rats, male | adult | 252-430 | Under urethane anesthesia | unilateral, left | 0.1 ms biphasic pulses, 200 ms train at 200 Hz, 100-500 µA, 250 ms prior to tone and juxtacellular current | Auditory cortex | single unit recording | stimulation in combination with auditory tone and juxtacellular electrical stimulation. 240 pairing trials, observation up to 30 mins post-conditioning | combined electrical stimulation of the NBM and local juxtacellular stimulation prevent plasticity of the auditory cortex |
| 14 | Kilgard et al 2001 ^49^ | Rats | NA | 300 g | awake state | NA | 20 pulses at 100 Hz, 100 us, 70-150 µA | Auditory cortex | microelectrode recording | 300-500 pairings per day for 4 weeks | The degree and direction of NBM-induced plasticity in the auditory cortex depends on the features of the auditory stimuli, such as the variability of tones and pulse presentation rate |
| 15 | Mercado 3rd et al 2001 ^50^ | rat, Sprague-Dawley, female | aduit | 300 g | awake state | unilateral, right | biphasic, 100 Hz, 100 us, 100 µA | auditory cortex | unit recording | NBM stimulation is presented duing the last 200 ms of tone presentation, 400 times for 20 days | cortical responses to sound can be powerfully, positively changed by experience with periodic FM sounds |
| 16 | McLin III et al, 2002 ^51^ | Sprague Dawley Rats, male | NA | 350-690 g | awake | unilateral, right | single burst of 200 ms, biphasic , 100 Hz, 200-us pulse width, 50–100 µA | Auditory cortex | local field potential recording at the auditory cortex, electrocardiography, respiratory analysis | 20 stimulation trials, effects observed up to 10 s post-stimulation | NBM stimulation induced autonomic-system-related behaviour that is normally engaged during learning: decreased theta and alpha power and increased gamma power in auditory cortex; biphasic heart rate changes; and disrupted ongoing respiration patterns |
| 17 | Kilgard & Merzenich 2002 ^52^ | rat | NA | NA | awake, freely moving | NA | biphasic, 100 Hz, 100 us, 70-140 µA | auditory cortex | unit recording | NBM stimulation is paired with tone presentation | context-dependent facilitation by basal forebrain activation is highly experience-dependent |
| 18 | McLinIII et al 2003 ^53^ | Sprague Dawley Rats, male | NA | 350-690 g | awake | unilateral | 0.2ms pulses,100Hz, 200ms, biphasic | Auditory cortex | local field potential recording at the auditory cortex | 15 days of pairing trials (200 trials/day) and 24-hr post-training measurement | Electrical NBM stimulation elicited conditioned response in forms of decreased tetha, and alpha oscillations as well as increased gamma activity during both training and testing of frequency generalization gradient |
| 19 | Ma & Suga 2003 ^54^ | Brown bats | adult | 18-24 g | Under innovar anesthesia | unilateral | continuous, 100 Hz, 200 us, 100 µA, 15-30 minutes | Auditory cortex, inferior colliculus | single unit recording | 15-30 minutes of basal forebrain stimulation combined with auditory cortex stimulation and somatosensory cortex stimulation | electrical NBM stimulation augments the frequency-tuning plasticity of the auditory cortex and of the inferior colliculus. The effect is larger in longer stimulation duration and when pairing simultaneously with electrical stimulation of the auditory cortex and the somatosensory cortex. |
| 20 | Zhang et al 2005 ^55^ | Mice, C57, female | 7-9 weeks | 18.3-25.9 g | under ketamine & xylazine anesthesia | unilateral, left | a 200-ms-long train of electrical pulses (200 us pulsewidth, monophasic square wave, 120 Hz), 67-132 µA | Auditory cortex, inferior colliculus | tone-evoked action potential recording | 360 stimulation trains over 6 minutes pre-conditioning, then during conditioning sessions by which it is paired with conditioned tone. The maximum plasticity effect was observed 30 minutes post-conditioning dissipated up to 240 minutes post stimulation | Electrical stimulation of the NB paired with a tone shifted the frequency tunings of inferioc colliculus neurons towards the frequency of the paired tone. The frequency shift is prevented by applying muscimol and atropine in the auditory cortex |
| 21 | Yan & Zhang et al 2005 ^56^ | Mice, C57/6J, female | 7-9 weeks | 19.6–24.6 g | Under ketamine-xylazine anesthesia | unilateral, left | 200-ms-long trains of 100-Hz monophasic pulses, 200 us, 83-175 µA | Auditory cortex | single unit recording | (i) ES_NB_ and tones were delivered randomly, (ii) ES_NB_ and tones were delivered synchronously with a 500‐ms ES_NB_ delay, (iii) ES_NB_ was delivered alone, i.e. 2nd ES_NB_ alone, and (iv) tone was delivered alone., (v) paired ESNB-tone. 1.5 hr ESNB stimulation, then 6 mins of pairing session. 60 ms tone and 200 ms ESNB | Both paired and unpaired electrical stimulation of the NBM and conditioned tone induces receptive field plasticity of the auditory cortex towards the frequency of the paired tone which reaches the change peak at 30 minutes after the pairing and gradually disappear up to 6 hrs post pairing. |
| 22 | Pandya et al 2005 ^57^ | rat, Sprague-Dawley, male | NA | NA | under awake state | unilateral, right | 20 biphasic pulses, 100 Hz, 100 us for 200 ms, 70-180 µA | auditory cortex | single-unit recording | tone-NBM-paired stimulation 300 times for 20 days | pairing nucleus basalis stimulation with 2 and 14 kHz tones results in the expansion of the receptive field in the primary auditory cortex to frequencies below 2 kHz and above 14 kHz, but cortical excitability was specifically decreases upon these frequencies |
| 23 | Moucha et al 2005 ^58^ | rat, Sprague-Dawley, female | NA | NA | under awake state | unilateral, right | 20 biphasic pulses, 100 Hz, 100 us for 200 ms, 70-180 µA | auditory cortex | single-unit recording | 4-8 kHz tone-NBM-paired stimulation 300 times for 20 days | Pairing tone with NB stimulation decreased tone thresholds, frequency selectivity, and response latency of A1 neurons in the region of the tonotopic map activated by the sound |
| 24 | Zhang et al 2006 ^59^ | M1-knockout and wild-type mice | 6-8 weeks | 17-23 g | Under ketamine-xylazine anesthesia | unilateral | 200-ms train, 120 Hz, 200 us, 76-141 µA | auditory cortex | EEG | tone paired with NBM stimulation | the shifting of the frequency tuning after tone-NBM stimulation pairing is significantly lower in M1-knockout mice compared to the wild types |
| 25 | Kilgard et al 2007 ^60^ | rat, Sprague-Dawley, female | adult | NA | awake state | unilateral, right | biphasic, 100 Hz, 100 us, 70-140 µA | auditory cortex | single-unit recording | tone paired with NBM stimulation | repetitive pairing of tones-nucleus basalis (NB) stimulation increases EEG synchronization and expands receptive field size for the specific training frequency while pairing tones of different stimulation frequency with NBM stimulation decreases the receptive field size and EEG synchronization |
| 26 | Pucket et al 2007 ^61^ | rat, Sprague-Dawley, female | adult | 250-350 g | awake state | unilateral, right | 20 biphasic pulses, 100 Hz, 100 us for 200 ms, 70-180 µA | posterior auditory field | single-unit recording | tone paired with NBM stimulation | NB–tone pairing elicits the frequency selectivity within the posterior auditory field and expands the receptive field of the area for the trained tone frequency |
| 27 | Chen & Yan 2007 ^62^ | C57BL/6 mice, female | 4-7 weeks | 17-21 g | under ketamine-xylazine anesthesia | unilateral | 200-ms train, 120 Hz, 200 us, 92-167 µA | auditory cortex | EEG | tone (10 dB below neuronal excitation threshold) paired with NBM stimulation | BF stimulation reduced the response threshold of the recorded cortical neurons to the frequency of the paired tone. Topical atropine administration eliminated the effect of BF activation |
| 28 | Zhang & Yan 2008 ^63^ | C57BL/6 mice, female | 5-6 weeks | 15-22 g | under ketamine-xylazine anesthesia | unilateral | 200-ms train, 120 Hz, 200 us, 92-167 µA | medial geniculate nucleus | multi-unit recording | tone paired with NBM stimulation | the paired tone-NBM stimulation elicited significant shifting of the MGB receptive fields toward the frequency of the paired tone. The receptive field reorganization is abolished with the inhibition of the auditory cortex with GABA-A agonist |
| 29 | Weinberger et al 2013 ^64^ | Sprague Dawley Rats, male | unsp. | 412 +- 28 g | awake | unilateral, right | 200-ms-long train of 100 Hz biphasic pulses, 200-us pulsewidth, 100 μA | Auditory cortex | local field potential recording at the auditory cortex | 3 days of pairing trials followed by 24-hr post-trial observation | the peak increase of gamma activity in classical associative conditioning involving electrical stimulation of the NBM may be a signature of the specific contents of memory |
| 30 | Bieszczad et al 2013 ^65^ | Sprague Dawley Rats, male | adult | 400 g | awake and under pentobarbital anesthesia | unilateral, right | 200-ms-long train of 100 Hz biphasic pulses, 200-us pulsewidth, 100 μA | primary auditory cortex | electrophysiological mapping, respiration index assessment | during 3-day acquisition trial, observation about 24-48 days after the behavioral testing | Electrical NBM stimulation paired with a tone at predertermined frequency induces expansion of tonotopic representation of certain frequency in auditory cortex which associates with the strongest memory of each individual animal |
| 31 | Sakata 2016 ^66^ | rat, Sprague-Dawley, male | NA | 267-357 g | Under urethane anesthesia | unilateral, right | 100 Hz, 200 μs duration, 50 μA | the medial geniculate body (MGB) and auditory cortex | single-unit recording | NBM stimulation is paired with auditory stimulation | BF stimulation increased power at gamma frequency in superficial layers of the MGB and the auditory cortex and reduced the onset response variability in both AC and MGB |
| **II. Studies related to motor and somatosensory system** | | | | | | | | | | | |
| 32 | Rasmusson & Dykes, 1988 ^67^ | cat, male & female | NA | 2-4.5 kg | under halothane anasthesia | bilateral | 4 pulses at 400 Hz, 40 us, 50-100 µA | somatosensory cortex | multiunit recording | during experiment, electrical pulses paired with cutaneous stimulation | the somatosensory evoked potentials were enhanced after 10-660 pairings |
| 33 | Tremblay et al 1990 ^68^ | cat, mongrel, male & female | adult | NA | under halothane anesthesia | NA | 4 pulses in 8-ms train, 100 us, 50 µA | somatosensory cortex | single unit recording | 10 trains of electrical stimulation paired with iontophoretic administration of glutatamate in the somatosensory cortex | The BF electrical stimulation enhance the responsiveness of 54% of sampled cortical neurons to iontophoretically administered glutamate, both in excitatory and inhibitory fashion |
| 34 | Webster et al 1991 ^69^ | racoons | young adult | 4.5 - 7.5 kg | under chlorelase anesthesia | unilateral | trains of 4 pulses, 500 us, 300 Hz,delivered at 2-s intervals | somatosensory cortex | multiunit activity | paired with tactile stimulation at the forepaw | the NBM input enhances the efficacy of cortical responses to cutaneous input |
| 35 | Howard III & Simons, 1994 ^70^ | Sprague Dawley Rats, male | NA | 225-300 gr | Under urethane anesthesia | unilateral | single 10-20-s train of monophasic-50-Hz pulses, 500 us pulsewidth, 1-1.5 mA | layer V of somatosensory cortex | microelectrode recording | effects was observed starting from 2 mins post stimulation up to two hours post stimulation | The response properties of somatosensory cortical neurons, such as the whisker-deflection-induced spike activity and the spontaneous bakcground activity were modulated differently by the electrical stimulation of the NBM, depending on factors such as the time interval between successive stimulations. The modulatory effects can be blocked by the administration of muscarinic antagonist, atropine, post-stimulation |
| 36 | Jimenez-Capdeville et al 1997 ^71^ | rat, Sprague-Dawley, male | NA | 200 g | Under urethane anesthesia | unilateral | 100 Hz, 200 us, 400-500 µA in a 100-500 ms trainThe applied current (400–500 µA) consisted of 0.2 ms pulses at 100 Hz (Rasmusson et al., 1992) for periods of 100–500 ms every 1-5 s | motor and somatosensory cortex | unit recording, microdyalisis | 30 minutes | while electrical stimulation at various sites of NBM can elicit cortical acetylcholine release, the cortical neuronal responses were varried, showing both excitatory and inhibitory effect of the BF stimulation |
| 37 | Wyrwicka & Chase 2001 ^72^ | cat | NA | NA | awake, freely moving cat | bilateral | 4-5 pulses at 100 Hz, 0.5-2 V for 100 ms | sensorimotor cortex | EEG | 30 minutes, 2 to 3 times per week | electrical stimulation to the basal forebrain area evoked a slow-wave EEG pattern and pre-sleep behavior pattern |
| 38 | Golmayo et al 2003 ^73^ | rat, Wistar, male & female | NA | NA | Under urethane anesthesia | NA | 100 Hz, 300 us, 20-200 µA | prefrontal, somatosensory, and visualcortex | EEG and unit recording | 200 ms per stimulation train | BF stimulation increased the amplitude of both visual- and somatosensory-evoked potentials. Atropine blocked both facilitatory effects. |
| 39 | Marino & Cudeiro 2003 ^74^ | cat, male & female | NA | NA | under α-chloralose anesthesia | NA | cathodic pulse, 50 Hz, 50 us, 0.1-1 mA | somatosensory, visual cortex | ECoG, unit recording, voltammetric recording | 2-4 s every 5-10 minutes | NOS activity significantly reduces the ability of BF stimulation to induce desynchronization of EEG pattern |
| 40 | Berg et al 2005 ^75^ | rats, Long Evans, female | NA | 200-300 g | awake animal | unilateral, right | monophasic, a train of 50 pulses at 500 Hz, 200 us pulse width, 100 µA. | M1 motor cortex, hipocampus | mystacial EMG, LFP | carryover effect up to 10 s, desynchronization effect observed for 3 s and dissipated. ICMS performed for 3 s (20 trials) | Electrical NBM stimulation elicits activation of cortically-projecting cholinergic neurons and strengthens vibrissae contraction induced by intracortical microstimulation of the M1. EEG desynchronization in M1 and hippocampus induced by NBM electrical stimulation. |
| 41 | Kuo et al 2009 ^76^ | Long-Evans rats, male | adult | 300-500 g | Under urethane anesthesia | unilateral, right | Intermittent, 0.5 s on and 10 s off, 200 us negative pulses, 1 mA, 100 Hz, 10 times | somatosensory/barel cortex, primary visual cortex | fPSP, multiunit recording, | 10 times of stimulation burst with 2 hr-observation post-stimulation | Electrical stimulation of the NBM potentiates neuronal activation of V1 and S1 cortex which receives non-dominant presynaptic input. |
| 42 | Takata et al 2011 ^77^ | Mice, C57BL/6 WT and IP_3_R2-KO, male mice | 8-12 wk old | NA | Under urethane anesthesia | unilateral, unsp. | a single train of 50 pulses at 100 Hz, 500 us pulsewidth, 200 μA | somatosensory cortex | multichannel extracellular recording, microdyalisis-HPLC for D-ser measurement, two-photon imaging | pairing whisker-NBM stimulation for 5 mins, observation up to 60 mins | Electrical NBM stimulation activates astrocytes to secrete NMDAR-coagonist D-serin which contributes to plasticity in somatosensory cortex |
| 43 | von Kraus & Francis 2014 ^78^ | rat | NA | NA | under pentobarbital anesthesia | unilateral | 30 biphasic pulses, 100 Hz, 400 µA | somatosensory cortex | multi-unit recording | NBM stimulation is either paired or unpaired with tactile stimulation | BF electrical stimulation paired with whisker deflectio causes an increase in the center-surround contrast of the treated whisker’s cortical response field |
| 44 | Espinosa et al 2015 ^79^ | Cats, male & female | adult |  | under isoflurane anesthesia | unilateral | rectangular cathodal, 50 Hz, 50 us, 0.1-1 mA for 2 s | somatosensory cortex | ECoG, light spectropscopy | during surgery | BF stimulation enhances the cortical level of nitric oxide in the cat cortex along with its effect in inducing desynchronization of cortical actitvity |
| 45 | Meir et al 2018 ^80^ | Mice, C57BL/6J | 9-16 weeks | NA | awake and under halothane anesthesia | unilateral | 50 pulses, 100 Hz, 500 us, 100-200 µA | barrel cortex | extracellular and whole-cell patch recordings | during surgery and recording | NBM stimulation enhances the signal-to-noise ratio of cortical sensory response by reducring the rate and amplitude of background synaptic activity and not by directly enhancing the reponse of sensory stimuli |
| 46 | Goshadrou & Sadeghi 2020 ^81^ | rat, Wistar, male | 3 months | 250-350 g | Under urethane anesthesia | bilateral | 15 pulses, 50 Hz, 200 us, 30 µA | layer V of somatosensory cortex | single-unit recording | periodic stimulation, 50 times during the recording trial | NBM electrical stimulation paired with vibrissal tactile stimulation decreased ON and OFF response magnitude in half of the observed units while the spontaneous firings remained unchanged |
| 47 | Vardar & Guclu 2020 ^82^ | rat, Wistar, male & female | adult | NA | Under ketamine-xylazine anesthesia | unilateral, left | intermittent (500 ms on/off cycle) biphasic, 100 Hz, 500 us 50 µA | layer III-VI of somatosensory cortex | single unit recording | periodic, during electrophysiological recording experiment | BF electrical stimulation increased the synchronization of neuronal response over time, mainly in layer III and VI of the primary somatosensory cortex, and when low-frequency (5-Hz) vibrotactile stimuli was applied |
| **III. Studies related to visual system** | | | | | | | | | | | |
| 48 | Hanganu et al 2007 ^83^ | rat | 5-6 days | NA | under hypothermic anesthesia | unilateral | single pulse: 5 V; 50 μs; 0.1 Hz or tetanic: 1–10 V; 10 pulses at 10 Hz for 1 s every 15 s | V1 cortex | multiple unit activity recording | acute stimulation during the experiment | BF stimulation increased the occurrence of V1 spindle bursts in neonatal V1 cortex |
| 49 | Dringenberg et al 2007 ^84^ | rat, Long-Evans, male | adult | 300-450 g | Under urethane anesthesia | unilateral | cathodic pulses, 100 Hz, 200 us, 500 µA in 0.5 s train for 10 times | visual cortex | fPSP recording, ECoG | NBM stimulation is paired with theta burst stimulation (TBS) of the lateral geniculate nucleus | Basal forebrain stimulation enhanced long term potentiation induced by weak and strong theta burst stimulation of the lateral geniculate nucleus when applied 5 minutes after the TBS but not to 30 minutes after and 5 minutes before TBS |
| 50 | Goard & Dan 2009 ^85^ | male, Long Evans, | 250-350 g | adult | Under urethane anesthesia | unilateral, left | trains of 50 pulses (0.1 ms per pulse) at 100 Hz | LGN, V1, visual cortex | multiunit recording, single unit recording | stimulation during the presence of visual stimuli (video) | Electrical stimulation of the NBM enhances the processing of visual stimuli by trigerring decorrelation of neuronal activities in the visual cortex and increases the reliability of neuronal responses across trials. |
| 51 | Gagolewicz & Dringenberg 2009 ^86^ | rat, Long-Evans, male | adult | 300-600 g | Under urethane anesthesia | unilateral | cathodic pulse, 10 pulses at 100 Hz, 200 us, 1 mA, repeated 50 times at 0.5 Hz | visual cortex | fPSP recording, ECoG | periodic pairing stimulation of the LGN and the NBM | the pairing of basal forebrain-lateral geniculate nucleus stimulation potetiate the fPSPs recorded in the contralateral V1 in response to stimulation of the contralateral LGN |
| 52 | Chen et al 2012 ^87^ | Mice, wild type and conditional inositol 1,4,5 trisphosphate receptor type KO (IP3R2-cKO) mice, C57BL/6 | 2-3 wk & >6wk old | - | under anesthesia (0.2–0.5% isoflurane or fentanyl/medetomidine) | unilateral, left | trains of 50 electrical pulses at 100 Hz, 100 us | the supragranular layer of the primary visual cortex | in vivo cell-atached recording, in vivo two-photon calcium imaging, ex vivo calcium imaging, | potentiation of V1 neuron response after pairing visual & NBM stimulation for about 40 minutes, calcium response of astrocytes post NBM stimulation was observed within 20-sec period | NBM electrical stimulation elicited stimulus-specific activity potentiation of neurons in the primary visual cortex and is mediated by the cholinergic activation of astrocytes via muscarinic receptors. |
| 53 | Alitto & Dan 2012 ^88^ | transgenic CaMKIIα+/PV+/VIP+/SOM+ fluorescent-tag mice | NA | NA | Under urethane anesthesia | unilateral, left | 100 Hz, 100 us | layers 1 and 2/3 of mouse visual cortex | two-photon calcium imaging, EEG | limited during surgery | electrical stimulation of the BF differentially modulates the activity of cortical excitatory and inhibitory neurons depending on the level of EEG desynchronization |
| 54 | Bhattacaryya et al 2013 ^89^ | tree shrew, *Tupaia belangeri* | 3-9 years | NA | under isoflurane anesthesia | unilateral | 500-ms train, anodic pulse, 100 Hz, 50 us, 7-10V (~10-20 µA) | visual cortex | single- and multi-unit activity | acute stimulation, paired with visual cues | BF stimulation increased V1 single and multi-unit activity as well as contrast sensitivity while the orientation selectivity decreases |
| 55 | Kimura et al 2014 ^90^ | VGAT-Venus or GAD67-GFP (Δneo) mice | 9-12 weeks | NA | Under urethane anesthesia | unilateral, left | 100 Hz, 500 us | primary visual cortex | two-photon imaging, whole-cell recording | 2-s train paired with visual cues | BF stimulation increased the magnitude and reliability of visual responses of GABAergic neurons whereas for the excitatory neurons, the decay of responses became faster following visual stimulation |
| 56 | De Luna et al 2017 ^91^ | tree shrew, Tupaia belangeri | 3-9 years | NA | under isoflurane anesthesia | unilateral | 500-ms train, anodic pulse, 100 Hz, 50 us, 7-10V (~10-20 µA) | primary visual cortex | local field potential recording | periodic within electrophysiological recording experiment | BF stimulation enhanced trial-to-trial reliability of LFP signals, occurred mainly within the low-frequency band of the LFP |
| **IV. Studies related to the role of the NBM electrical stimulation on biological functioning and therapy** | | | | | | | | | | | |
| 57 | Lineberry et al 1971 ^92^ | cats | NA | NA | awake mobile and restrained state | bilateral | 5 Hz, 100 us, 0.4-2 mA | frontal and occipital cortices | EEG, unit recording | during behavioural and neurophysiological recording | BF stimulation elicit behavioural inhibition, EEG synchronization and modulation of unit activity in the mesencephalic tegmentum |
| 58 | Siegel et al 1986 ^93^ | cats | NA | NA | under pentobarbital anesthesia | bilateral | Intermittent, 50 ms trains of 100 Hz, 100 us, 6 cycles/second, 285-950 µA | the pericruciate cortex and posterior lateral gyrus | EEG | during experiment | The degree of spinal inhibition by BF stimulation decreased from caudal to rostral regions. |
| 59 | McLachlan & Bihari 1990 ^94^ | rat, Wistar | NA | 200-350 g | Under urethane anesthesia | unilateral, left | biphasic, 40-50 Hz, 200 us, .4-0.8 µA | frontal parietal cortex | EEG, EMG | during experiment, 2-15 s during the presence of cortical epileptical focus induced by penicillin | The BF stimulation results in cortically diffused, bihemispheric, theta rhythm which never outlasts beyond the stimulation period and appears only after the application of peniciline. The NBM-induced EEG synchronization is followed by generalized seizure. |
| 60 | Sinnamon 1992 ^95^ | rat, Sprague-Dawley | NA | 250-500 g | under nembutal anesthesia | NA | cathodal pulse, 50 Hz, 500 us, 25-50 µA | hindlimb muscle | EMG | 10 s per stimulation site | stimulation to the NBM do not elicit locomotion |
| 61 | Blik et al 2015 ^96^ | Rats, WAG/Rij | NA | NA | awake state | NA | 100-150 Hz, 100 us | frontal cortex | adaptive DBS | 1-s NBM stimulation when spike-wave discharges detected | adaptive DBS of the NBM desynchronizes the epileptiform activity as detected by EEG |
| 62 | Cherian et al 2017 ^97^ | rat, Sprague-Dawley, male | 3 months | NA | Under urethane anesthesia | unilateral, right | Intermittent (500 ms on/off cycle), 100 us, 100 μA | frontal cortex and striatum | synaptosome analysis | 20 minutes | BF stimulation increased cortical choline transporter (CHT)-mediated choline transport in rats with reward-oriented behavior while remained unchanged in animals which were incentive-salience-oriented |
| 63 | Yamakawa et al 2016 ^98^ | Syrian hamster, male | NA | NA | awake state | unilateral | biphasic pulse, 50 Hz, 500 us, 200 µA | suprachiasmatic nucleus | actimetry | periodic during behavioral assessment | electrical stimulation of the basal forebrain shifts the circadian clock and these shifts can be prevented by infusions of atropine to the SCN |
| **V. Studies exploring connetivity between the NBM and cortical-subcortical structures** | | | | | | | | | | | |
| 64 | Inoue et al 1983 ^99^ | Macaca mulatta, Macaca fuscata | NA | 4-6 kg | awake state | unilateral, unsp. | 0.3 Hz, 200 us pulsewidth, 0.2-0.8 mA | dorsolateral PFC | single unit recording | immediate effect within 1 sec post stimulation | electrical NBM stimulation elicites cholinergic neuron activation and excitates a proportion of neurons in the dorsolateral prefrontal cortex |
| 65 | Abdulla et al 1994 ^100^ | Rats, Sprague-Dawley, male | NA | 200-250 g | Under urethane anesthesia | unilateral | monophasic, single pulse, 300 µA, 500 us | frontal cortex | unit activity recording | single pulse, during recording | Single-pulse NBM stimulation elicited both excitatory or inhibitory effects frontal cortical neurons |
| 66 | Racine et al 1995 ^101^ | Rats, Long Evans, male | NA | 350-550 g | under ketamine/xylazine anesthesia | unilateral | biphasic, 10 Hz, 100us, 500 µA, paired with callosal stimulation | frontal cortex | EEG | during recording | When paired with electrical stimulation of corpus callosum, acute NBM-ES induced neocortical long term depression |
| 67 | Nguyen & Lin 2014 ^102^ | rat, Long Evans | 3-6 months | NA | under isoflurane anesthesia and awake state | bilateral | biphasic pulse, 0.5 Hz, 100 us | frontal cortex | EEG | periodic, during recording | BF electrical stimulation in the absence of any auditory stimulus elicited highly reliable LFP responses in frontal cortical regions |
| 68 | Nagasaka et al 2017 ^103^ | rat, Sprague-Dawley, male | NA | 220-380 g | under urethane anesthesia | unilateral | 1 Hz, 300 µA, 150 µA | frontal cortex | Voltage-sensitive-dye imaging | periodic stimulation within electrophysiological recording experiment | the anteroposterior axis of the NBM projected to the mediolateral axis of the dorsal frontal cortex |
| 69 | Mancia et al 1976 ^104^ | cat | adult | NA | under anesthesia | NA | 6-8 Hz, 60-100 Hz | brain stem | Intracellular recording | during surgery | BF stimulation induced excitation of bulbar and caudopontine neurons and in less extent, neurons in the rostropontine and mesencephalic neurons. |
| 70 | Femano et al, 1983 ^105^ | Cat | unsp. | 2.5-3.8 kg | Under ketamine anesthesia | unilateral | monophasic, 0.2-Hz cathodal-square wave pulse, 500 us pulse width, 4µA-1000 µA | Amygdala | extracellular unit activity recording | excitation pattern immediately post-stimulation | the anatomical connectivity between the lateral aspect of the substantia innominata (nucleus basalis) and the amygdala is shown |
| 71 | Szymusiak & McGinty, 1989 ^106^ | cat | NA | NA | under anesthesia | unilateral | monophasic, 1 Hz, 200 us, 0.4-1.2 mA | midbrain reticular formation | unit recording | during recording, surgery | electrical stimulation at the medial and lateral basal forebrain induces brief excitation followed by a longer period of discharge suppression. The suppression duration was longest when the lateral SI is stimulated |
| 72 | Mello et al 1992 ^107^ | Rats, Sprague-Dawley, male | NA | NA | under chloral hydrate anesthesia | NA | 0.5 Hz, 200 us, 30-800 µA | Amygdala | Extracellular and intracelluar recording | during surgery and recording | 80% of recorded amygdaloid neurons, both excitatoy and inhibitory neurons, were responsive to NBM stimulation |
| 73 | Kolmac & Mitrofanis 2000 ^108^ | rat, Sprague-Dawley | adult | 250-300 g | under halothane anesthesia | unilateral | intermittent (5 s on/off), 20-30 Hz, 200 µA | thalamic reticular nucleus (Rt) and zona incerta (ZI) | c-FOS immunohistochemistry | 20 minutes | c-FOS immunoreactive cells in the rostral pole of the Rt and rostral sector of the ZI are present |

**REFERENCES**

1. Casamenti, F., Deffenu, G., Abbamondi, A. L. & Pepeu, G. Changes in cortical acetylcholine output induced by modulation of the nucleus basalis. Brain Res. Bull. 16, 689–695 (1986).

2. Kurosawa, M. et al. Stimulation of the nucleus basalis of Meynert increases acetylcholine release in the cerebral cortex in rats. Neurosci. Lett. 98, 45–50 (1989).

3. Kurosawa, M., Sato, A. & Sato, Y. Well-maintained responses of acetylcholine release and blood flow in the cerebral cortex to focal electrical stimulation of the nucleus basalis of Meynert in aged rats. Neurosci. Lett. 100, 198–202 (1989).

4. Rasmusson, D.D., Clow, K., Szerb, J. C. Frequency-dependent increase in cortical acetylcholine release evoked by stimulation of the nucleus basalis magnocellularis in the rat. Brain Res. 594, 150–154 (1992).

5. Biesold, D., Inamami, O., Sato, A., & Sato, Y. Stimulation of the nucleus basalis of Meynert increases cerebral cortical blood flow in rats. Neurosci. Lett. 98, 39–44 (1989).

6. Adachi, T., Inanami, O., Ohno, K. & Sato, A. Responses of regional cerebral blood flow following focal electrical stimulation of the nucleus basalis of Meynert and the medial septum using the [14C]iodoantipyrine method in rats. Neurosci. Lett. 112, 263–268 (1990).

7. Hallström, A., Sato, A., Sato, Y. & Ungerstedt, U. Effect of stimulation of the nucleus basalis of Meynert on blood flow and extracellular lactate in the cerebral cortex with special reference to the effect of noxious stimulation of skin and hypoxia. Neurosci. Lett. 116, 227–232 (1990).

8. Adachi, T., Biesold, D., Inanami, O. & Sato, A. Stimulation of the nucleus basalis of Meynert and substantia innominata produces widespread increases in cerebral blood flow in the frontal, parietal and occipital cortices. Brain Res. 514, 163–166 (1990).

9. Kimura, A., Sato, A. & Takano, Y. Stimulation of the nucleus basalis of Meynert does not influence glucose utilization of the cerebral cortex in anesthetized rats. Neurosci. Lett. 119, 101–104 (1990).

10. Sato, A. & Sato, Y. Cerebral cortical vasodilatation in response to stimulation of cholinergic fibres originating in the nucleus basalis of Meynert. J. Auton. Nerv. Syst. 30 Suppl, S137-40 (1990).

11. Linville, D. G. & Arneric, S. P. Cortical cerebral blood flow governed by the basal forebrain: age-related impairments. Neurobiol. Aging 12, 503–510 (1991).

12. Adachi, T., Baramidze, D. G., & Sato, A. Stimulation of the nucleus basalis of Meynert increases cortical cerebral blood flow without influencing diameter of the pial artery in rats. Neurosci. Lett. 143, 173–176 (1992).

13. Adachi, T., Inanami, O. & Sato, A. Nitric oxide (NO) is involved in increased cerebral cortical blood flow following stimulation of the nucleus basalis of Meynert in anesthetized rats. Neurosci. Lett. 139, 201–204 (1992).

14. Raszkiewicz, J. L., Linville, D. G., Kerwinm J. F., Wagernaar, F., & Arneric, S. P. Nitric oxide synthase is critical in mediating basal forebrain regulation of cortical cerebral circulation. J. Neurosci. Res. 33, 129–135 (1992).

15. Linville, D. G., Giacobini, E. & Arneric, S. P. Heptyl-physostigmine enhances basal forebrain control of cortical cerebral blood flow. J. Neurosci. Res. 31, 573–577 (1992).

16. Linville, D. G., Williams, S., Arneric, S. P. Basal forebrain control of cortical cerebral blood flow is independent of local cortical neurons. Brain Res. 622, 26–34 (1993).

17. Linville, D.G., Wiliams, S., Raszkiewicz, J. L., Arneric, S. P. Nicotinic agonists modulate basal forebrain control of cortical cerebral blood flow in anesthetized rats. J. Pharmacol. Exp. Ther. 267, 440–448 (1993).

18. Sercombe, R., Lacombe, P., Springhetti, V., MacKenzie, E. T. & Seylaz, J. Basal forebrain control of cortical blood flow and tissue gases in conscious aged rats. Brain Res. 662, 155–164 (1994).

19. Vaucher, E., Borredon, J., Seylaz, J. & Lacombe, P. Autoradiographic distribution of cerebral blood flow increases elicited by stimulation of the nucleus basalis magnocellularis in the unanesthetized rat. Brain Res. 691, 57–68 (1995).

20. Zhang, F., Xu, S. & Iadecola, C. Role of nitric oxide and acetylcholine in neocortical hyperemia elicited by basal forebrain stimulation: evidence for an involvement of endothelial nitric oxide. Neuroscience 69, 1195–1204 (1995).

21. Iadecola, C. & Zhang, F. Permissive and obligatory roles of NO in cerebrovascular responses to hypercapnia and acetylcholine. Am. J. Physiol. 271, R990-1001 (1996).

22. Lacombe, P., Sercombe, R., Vaucher, E. & Seylaz, J. Reduced cortical vasodilatory response to stimulation of the nucleus basalis of Meynert in the aged rat and evidence for a control of the cerebral circulation. Ann. N. Y. Acad. Sci. 826, 410–415 (1997).

23. Vaucher, E., Borredon, J., Bonvento, G., Seylaz, J. & Lacombe, P. Autoradiographic evidence for flow-metabolism uncoupling during stimulation of the nucleus basalis of Meynert in the conscious rat. J. Cereb. Blood Flow Metab. 17, 686–694 (1997).

24. Uchida, S., Kagitani, F., Suzuki, A. & Aikawa, Y. Effect of acupuncture-like stimulation on cortical cerebral blood flow in anesthetized rats. Jpn. J. Physiol. 50, 495–507 (2000).

25. Hotta, H., Uchida S., Kagitani, F. Effects of stimulating the nucleus basalis of Meynert on blood flow and delayed neuronal death following transient ischemia in the rat cerebral cortex. Jpn. J. Physiol. 52, 383–393 (2002).

26. Hotta, H., Kanai, C., Uchida, S. & Kanda, K. Stimulation of the nucleus basalis of Meynert increases diameter of the parenchymal blood vessels in the rat cerebral cortex. Neurosci. Lett. 358, 103–106 (2004).

27. Seigneur, J., Kroeger, D., Nita, D. A. & Amzica, F. Cholinergic action on cortical glial cells in vivo. Cereb. Cortex 16, 655–668 (2006).

28. Hotta, H., Uchida S., Kagitani, F. Stimulation of the nucleus basalis of Meynert produces an increase in the extracellular release of nerve growth factor in the rat cerebral cortex. J. Physiol. Sci. 57, 383–387 (2007).

29. Hotta, H. Uchida, S., Shiba, K. Cerebral cortical blood flow response during basal forebrain stimulation in cats. Neuroreport 18, 809–812 (2007).

30. Kocharyan, A., Fernandes, P., Tong, X.-K., Vaucher, E. & Hamel, E. GABA released from specific subtypes of activated cortical interneurons contributes to the hemodynamic response to basal forebrain stimulation. J. Cereb. Blood Flow Metab. 27, BP05-01H (2007).

31. Hotta, H., Kagitani, F., Kondo, M. & Uchida, S. Basal forebrain stimulation induces NGF secretion in ipsilateral parietal cortex via nicotinic receptor activation in adult, but not aged rats. Neurosci. Res. 63, 122–8 (2009).

32. Hotta, H., Uchida, S., Kagitani, F. & Maruyama, N. Control of cerebral cortical blood flow by stimulation of basal forebrain cholinergic areas in mice. J. Physiol. Sci. 61, 201–209 (2011).

33. Uchida, S., Hotta, H., Misawa, H. & Kawashima, K. Sustained subcutaneous infusion of nicotine enhances cholinergic vasodilation in the cerebral cortex induced by stimulation of the nucleus basalis of Meynert in rats. Eur. J. Pharmacol. 654, 235–240 (2011).

34. Takata, N. et al. Cerebral blood flow modulation by Basal forebrain or whisker stimulation can occur independently of large cytosolic Ca2+ signaling in astrocytes. PLoS One 8, e66525 (2013).

35. Hotta, H. et al. Layer-specific dilation of penetrating arteries induced by stimulation of the nucleus basalis of Meynert in the mouse frontal cortex. J. Cereb. Blood Flow Metab. 33, 1440–1447 (2013).

36. Koch, M. & Ebert, U. Enhancement of the acoustic startle response by stimulation of an excitatory pathway from the central amygdala/basal nucleus of Meynert to the pontine reticular formation. Exp. Brain Res. 93, 231–241 (1993).

37. Metherate, R. & Ashe, J. H. Ionic flux contributions to neocortical slow waves and nucleus basalis-mediated activation: whole-cell recordings in vivo. J. Neurosci. 13, 5312–5323 (1993).

38. Metherate, R. & Ashe, J. H. Nucleus basalis stimulation facilitates thalamocortical synaptic transmission in the rat auditory cortex. Synapse 14, 132–143 (1993).

39. Hars, B., Maho, C., Edeline, J. M. & Hennevin, E. Basal forebrain stimulation facilitates tone-evoked responses in the auditory cortex of awake rat. Neuroscience 56, 61–74 (1993).

40. Edeline, J. M., Hars, B., Maho, C. & Hennevin, E. Transient and prolonged facilitation of tone-evoked responses induced by basal forebrain stimulations in the rat auditory cortex. Exp. brain Res. 97, 373–386 (1994).

41. Bakin, J. S. & Weinberger, N. M. Induction of a physiological memory in the cerebral cortex by stimulation of the nucleus basalis. Proc. Natl. Acad. Sci. 93, 11219–11224 (1996).

42. Brett, B. & Barth, D. S. Subcortical modulation of high-frequency (gamma band) oscillating potentials in auditory cortex. J. Neurophysiol. 78, 573–581 (1997).

43. Mercado, E. 3rd, Myers, C. E. & Gluck, M. A. A computational model of mechanisms controlling experience-dependent reorganization of representational maps in auditory cortex. Cogn. Affect. Behav. Neurosci. 1, 37–55 (2001).

44. Bjordahl, T. S., Dimyan, M. A. & Weinberger, N. M. Induction of long-term receptive field plasticity in the auditory cortex of the waking guinea pig by stimulation of the nucleus basalis. Behav. Neurosci. 112, 467–479 (1998).

45. Kilgard, M. P. & Merzenich, M. M. Plasticity of temporal information processing in the primary auditory cortex. Nature Neuroscience vol. 1 727–731 (1998).

46. Dimyan, M. A. & Weinberger, N. M. Basal forebrain stimulation induces discriminative receptive field plasticity in the auditory cortex. Behav. Neurosci. 113, 691–702 (1999).

47. Miasnikov, A. A., McLin, D. 3rd & Weinberger, N. M. Muscarinic dependence of nucleus basalis induced conditioned receptive field plasticity. Neuroreport 12, 1537–1542 (2001).

48. Cruikshank, S. J. & Weinberger, N. M. In vivo Hebbian and basal forebrain stimulation treatment in morphologically identified auditory cortical cells. Brain Res. 891, 78–93 (2001).

49. Kilgard, M. P. et al. Sensory input directs spatial and temporal plasticity in primary auditory cortex. J. Neurophysiol. 86, 326–338 (2001).

50. Mercado, E., Bao, S., Orduña I., Gluck, M. A., Merzenich, M. M. Basal forebrain stimulation changes cortical sensitivities to complex sound. Neuroreport 12, 2283–2287 (2001).

51. McLin, D. E. 3rd, Miasnikov, A. A. & Weinberger, N. M. The effects of electrical stimulation of the nucleus basalis on the electroencephalogram, heart rate, and respiration. Behav. Neurosci. 116, 795–806 (2002).

52. Kilgard, M. P., Pandya, P. K., Engineer, N. D. & Moucha, R. Cortical network reorganization guided by sensory input features. Biol. Cybern. 87, 333–343 (2002).

53. McLin III, D. E., Miasnikov, A. A. & Weinberger, N. M. CS-specific gamma, theta, and alpha EEG activity detected in stimulus generalization following induction of behavioral memory by stimulation of the nucleus basalis. Neurobiol. Learn. Mem. 79, 152–176 (2003).

54. Ma, X. & Suga, N. Augmentation of plasticity of the central auditory system by the basal forebrain and/or somatosensory cortex. J. Neurophysiol. 89, 90–103 (2003).

55. Zhang, Y., Hakes, J. J., Bonfield, S. P. & Yan, J. Corticofugal feedback for auditory midbrain plasticity elicited by tones and electrical stimulation of basal forebrain in mice. Eur. J. Neurosci. 22, 871–879 (2005).

56. Yan, J. & Zhang, Y. Sound-guided shaping of the receptive field in the mouse auditory cortex by basal forebrain activation. Eur. J. Neurosci. 21, 563–576 (2005).

57. Pandya, P. K. et al. Asynchronous inputs alter excitability, spike timing, and topography in primary auditory cortex. Hear. Res. 203, 10–20 (2005).

58. Moucha, R., Pandya, P. K., Engineer, N. D., Rathbun, D. L. & Kilgard, M. P. Background sounds contribute to spectrotemporal plasticity in primary auditory cortex. Exp. brain Res. 162, 417–427 (2005).

59. Zhang, Y., Hamilton, S. Nathanson, N. M., Yan, J. Decreased input-specific plasticity of the auditory cortex in mice lacking M1 muscarinic acetylcholine receptors. Cereb. Cortex 16, 1258–1265 (2006).

60. Kilgard, M. P., Vazquez, J. L., Engineer, N. D. & Pandya, P. K. Experience dependent plasticity alters cortical synchronization. Hear. Res. 229, 171–179 (2007).

61. Puckett, A. C., Pandya, P. K., Moucha, R., Dai, W. & Kilgard, M. P. Plasticity in the rat posterior auditory field following nucleus basalis stimulation. J. Neurophysiol. 98, 253–265 (2007).

62. Chen, G. & Yan, J. Cholinergic modulation incorporated with a tone presentation induces frequency-specific threshold decreases in the auditory cortex of the mouse. Eur. J. Neurosci. 25, 1793–1803 (2007).

63. Zhang, Y. & Yan, J. Corticothalamic feedback for sound-specific plasticity of auditory thalamic neurons elicited by tones paired with basal forebrain stimulation. Cereb. Cortex 18, 1521–1528 (2008).

64. Weinberger, N. M., Miasnikov, A. A., Bieszczad, K. M. & Chen, J. C. Gamma band plasticity in sensory cortex is a signature of the strongest memory rather than memory of the training stimulus. Neurobiol. Learn. Mem. 104, 49–63 (2013).

65. Bieszczad, K.M., Miasnikov, A. A. & Weinberger, N. M. Remodeling sensory cortical maps implants specific behavioral memory. Neuroscience 246, 40–51 (2013).

66. Sakata, S. State-dependent and cell type-specific temporal processing in auditory thalamocortical circuit. Sci. Rep. 6, 18873 (2016).

67. Rasmusson, D. D. & Dykes, R. W. Long-term enhancement of evoked potentials in cat somatosensory cortex produced by co-activation of the basal forebrain and cutaneous receptors. Exp. Brain Res. 70, 276–286 (1988).

68. Tremblay, N., Warren, R. A. & Dykes, R. W. Electrophysiological studies of acetylcholine and the role of the basal forebrain in the somatosensory cortex of the cat. I. Cortical neurons excited by glutamate. J. Neurophysiol. 64, 1199–1211 (1990).

69. Webster, H. H. et al. Long-term enhancement of evoked potentials in raccoon somatosensory cortex following co-activation of the nucleus basalis of Meynert complex and cutaneous receptors. Brain Res. 545, 292–296 (1991).

70. Howard, M. A. 3rd, Simons, D. J., Howard III, M. A. & Simons, D. J. Physiologic effects of nucleus basalis magnocellularis stimulation on rat barrel cortex neurons. Exp. Brain Res. 102, 21–33 (1994).

71. Jimenez-Capdeville, M. E., Dykes, R. W. & Myasnikov, A. A. Differential control of cortical activity by the basal forebrain in rats: a role for both cholinergic and inhibitory influences. J. Comp. Neurol. 381, 53–67 (1997).

72. Wyrwicka, W. & Chase, M. H. Importance of the environment in conditioned behavior. Physiol. Behav. 73, 493–497 (2001).

73. Golmayo, L., Nunez, A. & Zaborszky, L. Electrophysiological evidence for the existence of a posterior cortical-prefrontal-basal forebrain circuitry in modulating sensory responses in visual and somatosensory rat cortical areas. Neuroscience 119, 597–609 (2003).

74. Marino, J. & Cudeiro, J. Nitric oxide-mediated cortical activation: a diffuse wake-up system. J. Neurosci. 23, 4299–4307 (2003).

75. Berg, R. W. Friedman B., Schroeder L. F., Kleinfeld, D. Activation of nucleus basalis facilitates cortical control of a brain stem motor program. J. Neurophysiol. 94, 699–711 (2005).

76. Kuo, M.-C., Rasmusson, D. D., Dringenberg, H. C. Input-selective potentiation and rebalancing of primary sensory cortex afferents by endogenous acetylcholine. Neuroscience 163, 430–441 (2009).

77. Takata, N. et al. Astrocyte calcium signaling transforms cholinergic modulation to cortical plasticity in vivo. J. Neurosci. 31, 18155–18165 (2011).

78. von Kraus, L. M. et al. Electronically induced contrast enhancement in whisker S1 cortical response fields. Conf. Proc. ... Annu. Int. Conf. IEEE Eng. Med. Biol. Soc. IEEE Eng. Med. Biol. Soc. Annu. Conf. 2014, 2601–2604 (2014).

79. Espinosa, N., Cudeiro, J. & Marino, J. Spectroscopic measurement of cortical nitric oxide release induced by ascending activation. Neuroscience 285, 303–311 (2015).

80. Meir, I., Katz, Y. & Lampl, I. Membrane Potential Correlates of Network Decorrelation and Improved SNR by Cholinergic Activation in the Somatosensory Cortex. J. Neurosci. 38, 10692–10708 (2018).

81. Goshadrou, F. & Sadeghi, B. Nucleus basalis of Meynert modulates signal processing in rat layer 5 somatosensory cortex but leads to memory impairment and tactile discrimination deficits following lesion. Behav. Brain Res. 386, 112608 (2020).

82. Vardar, B. & Güçlü, B. Effects of basal forebrain stimulation on the vibrotactile responses of neurons from the hindpaw representation in the rat SI cortex. Brain Struct. Funct. 225, 1761–1776 (2020).

83. Hanganu, I. L., Staiger, J. F., Ben-Ari, Y. & Khazipov, R. Cholinergic modulation of spindle bursts in the neonatal rat visual cortex in vivo. J. Neurosci. 27, 5694–5705 (2007).

84. Dringenberg, H. C., Hamze, B., Wilson, A., Speechley, W. & Kuo, M.-C. Heterosynaptic facilitation of in vivo thalamocortical long-term potentiation in the adult rat visual cortex by acetylcholine. Cereb. Cortex 17, 839–848 (2007).

85. Goard, M. & Dan, Y. Basal forebrain activation enhances cortical coding of natural scenes. Nat. Neurosci. 12, 1444–1449 (2009).

86. Gagolewicz, P. J. & Dringenberg, H. C. Selective potentiation of crossed vs. uncrossed inputs from lateral geniculate nucleus to visual cortex by the basal forebrain: potential facilitation of rodent binocularity. Neurosci. Lett. 463, 130–134 (2009).

87. Chen, N. et al. Nucleus basalis-enabled stimulus-specific plasticity in the visual cortex is mediated by astrocytes. Proc. Natl. Acad. Sci. U. S. A. 109, E2832-41 (2012).

88. Alitto, H. J. & Dan, Y. Cell-type-specific modulation of neocortical activity by basal forebrain input. Front. Syst. Neurosci. 6, 79 (2012).

89. Bhattacharyya, A., Veit, J., Kretz, R. Bondar, I. & Rainer, G. Basal forebrain activation controls contrast sensitivity in primary visual cortex. BMC Neurosci. 14, 55 (2013).

90. Kimura, R. et al. Curtailing effect of awakening on visual responses of cortical neurons by cholinergic activation of inhibitory circuits. J. Neurosci. 34, 10122–10133 (2014).

91. De Luna, P. et al. Basal forebrain activation enhances between-trial reliability of low-frequency local field potentials (LFP) and spiking activity in tree shrew primary visual cortex (V1). Brain Struct. Funct. 222, 4239–4252 (2017).

92. Lineberry, C.G. & Siegel, J. EEG synchronization, behavioral inhibition, and mesencephalic unit effects produced by stimulation of orbital cortex, basal forebrain and caudate nucleus. Brain Res. 34, 143–161 (1971).

93. Siegel, J., Morton, C. R., Sandkuhler, J., Xiao, H. M. & Zimmermann, M. Spinal neuronal inhibition and EEG synchrony by electrical stimulation in subcortical forebrain regions of the cat. Exp. brain Res. 62, 363–372 (1986).

94. McLachlan, R. S. & Bihari, F. Secondary generalization of seizures from a cortical penicillin focus following stimulation of the basal forebrain. Exp. Neurol. 109, 237–242 (1990).

95. Sinnamon, H. M. Microstimulation mapping of the basal forebrain in the anesthetized rat: The ‘preoptic locomotor region’. Neuroscience 50, 197–207 (1992).

96. Blik, V.A., Aristov, A.V. & Chepurnova, N.E. The Use of Automated System for EEG Analysis and Feedback Cerebral Stimulation to Stop Epileptiform Activity in WAG/Rij Rats. Bull. Exp. Biol. Med. 158, 520–522 (2015).

97. Koshy Cherian, A. et al. Unresponsive Choline Transporter as a Trait Neuromarker and a Causal Mediator of Bottom-Up Attentional Biases. J. Neurosci. 37, 2947–2959 (2017).

98. Yamakawa, G. R. et al. The cholinergic forebrain arousal system acts directly on the circadian pacemaker. Proc. Natl. Acad. Sci. U. S. A. 113, 13498–13503 (2016).

99. Inoue, M. et al. Cholinergic role in monkey dorsolateral prefrontal cortex during bar-press feeding behavior. Brain Res. 278, 185–194 (1983).

100. Abdulla, F. A. et al. An iontophoretic study of the effects of alpha-amino-hydroxy-5-methyl-4-isoxazole propionic acid lesions of the nucleus basalis magnocellularis on cholinergic and GABAergic influences on frontal cortex neurones of rats. Exp. brain Res. 98, 441–456 (1994).

101. Racine, R. J., Chapman, C. A., Trepel, C., Teskey, G. C. & Milgram, N. W. Post-activation potentiation in the neocortex. IV. Multiple sessions required for induction of long-term potentiation in the chronic preparation. Brain Res. 702, 87–93 (1995).

102. Nguyen, D. P. et al. A frontal cortex event-related potential driven by the basal forebrain. Elife 2014, e02148 (2014).

103. Nagasaka, K. et al. Topographical projections from the nucleus basalis magnocellularis (Meynert) to the frontal cortex: A voltage-sensitive dye imaging study in rats. Brain Stimul. 10, 977–980 (2017).

104. Mancia, M. et al. Basal forebrain and hypothalamic influences upon brain stem neurons. Brain Res. 107, 487–497 (1976).

105. Femano, P. A., Edinger, H. M., Siegel, A. The effects of stimulation of substantia innominata and sensory receiving areas of the forebrain upon the activity of neurons within the amygdala of the anesthetized cat. Brain Res. 269, 119–132 (1983).

106. Szymusiak, R. et al. Effects of basal forebrain stimulation on the waking discharge of neurons in the midbrain reticular formation of cats. Brain Res. 498, 355–359 (1989).

107. Mello, L. E., Tan, A. M. & Finch, D. M. Convergence of projections from the rat hippocampal formation, medial geniculate and basal forebrain onto single amygdaloid neurons: an in vivo extra- and intracellular electrophysiological study. Brain Res. 587, 24–40 (1992).

108. Kolmac, C. & Mitrofanis, J. Induction of Fos-like immunoreactivity in the ventral thalamus after electrical or chemical stimulation of various subcortical centres of rats. Neurosci. Lett. 301, 195–198 (2001).
